# Identifying and alleviating ethical risks of artificial intelligence in physical education: a systematic review

**DOI:** 10.3389/fpubh.2026.1945681

**Published:** 2026-09-10

**Authors:** Shun Chen, Chaojun Zhang, Quanxian Wang

**Affiliations:** School of Physical Education, Wuhan Sports University, Wuhan, China

**Keywords:** artificial intelligence, physical education, health promotion, technology ethics, educational ethics, body ethics

## Abstract

**Background:**

Physical education is a key public health setting for promoting physical fitness and lifelong healthy behaviors among children and adolescents. However, ethically inappropriate applications of artificial intelligence in physical education (AIPE) may undermine students’ bodily autonomy, educational equity, and the quality of their participation in health-promoting activities. This study aims to systematically identify the ethical risks associated with AIPE, analyze their causes and potential harms, and integrate targeted alleviation strategies.

**Methods:**

Following the PRISMA guidelines, a systematic search was conducted across six databases: SpringerLink, Web of Science, EBSCOhost, ScienceDirect, Scopus, and CNKI. After multiple rounds of screening, a total of 92 studies published in English or Chinese between January 2016 and May 2026 were included. A hybrid deductive-inductive thematic analysis was employed, with two researchers independently coding the included studies and cross-checking their results to extract and synthesize the types of ethical risks associated with AIPE and the corresponding alleviation strategies.

**Results:**

Ethical risks in the technology dimension include data leakage, privacy infringement, algorithmic bias, and algorithmic limitations. Risks in the physical education dimension include homogeneous physical education teaching, threats to teachers’ professional roles and agency, deviation from the goals of physical education, homogenization of student development, alienation in teacher-student relationships, alienation in student–student relationships, lack of humanistic care, value alienation, and academic misconduct. Risks in the body dimension include blurred body boundary, body meaning deconstruction, and body value alienation. Based on the analysis of the types, causes, and potential harms of these risks, this study adopts a stakeholder perspective to develop a systematic set of alleviation strategies encompassing three key dimensions: technological governance, educational regulation, and body protection.

**Conclusion:**

Achieving trustworthy AIPE requires balancing technological reliability, appropriateness for physical education, and the preservation of human agency. This study provides a theoretical reference for the ethical governance of AIPE within the field of public health. Future research should strengthen empirical validation, foster international cooperation, and advance research on artificial intelligence ethics.

**Systematic review registration:**

https://www.crd.york.ac.uk/PROSPERO/view/CRD420261338255, identifier PROSPERO (CRD420261338255).

## 1 Introduction

Artificial intelligence (AI) is increasingly being integrated into physical education, with its applications expanding in both scope and depth (1). Artificial intelligence in physical education (AIPE) can provide personalized feedback and dynamic adjustments based on students’ individual needs and athletic abilities, thereby enabling students to receive physical education programs tailored to their developmental stage and personal characteristics (2–4). Specifically, using smart wearable devices, computer vision, and virtual simulation technologies, it monitors data in real time, corrects movements and provides personalized guidance, thereby helping students develop their knowledge and skills in physical education. These applications not only optimize physical education teaching processes but also facilitate innovation in instructional approaches, thereby promoting educational equity by enabling personalized instruction within large-scale teaching contexts. Meanwhile, generative artificial intelligence (GAI) has achieved significant success in recent years, enabling users to create text, images, videos and even computer code, while offering insights that traditional tools cannot provide (5). Integrating diverse learning components and adjusting GAI implementation parameters can optimize its effectiveness in enhancing learning outcomes across diverse levels of education (6).

However, the empowering effects of technology are invariably accompanied by latent or manifest ethical risks, and the application of AIPE is by no means value-neutral and may pose a threat to public health. Firstly, there is the risk of algorithmic bias (7), which may lead to unfair assessments of students’ physical fitness, skills and even potential, as well as unequal allocation of resources, and may even reinforce discriminatory practices in new forms. Secondly, the large-scale collection of biometric, performance and behavioral data raise concerns regarding privacy infringement, data security (8) and informed consent. Agent systems may inadvertently store, disseminate and re-expose private or confidential information across tasks, users and sessions through long-term memory modules, vector databases, execution logs and feedback loops (9). Furthermore, over-reliance on technological quantitative metrics may undermine teachers’ agency, alienate teacher-student interactions, and narrow the understanding of the diverse educational values of physical education. These potential or already manifested ethical risks may generate a crisis of trust among stakeholders in physical education settings regarding the use of artificial intelligence. How can the field move toward trustworthy AIPE? This question constitutes the central concern of the present study.

Although previous studies have examined the ethical risks of AIPE, existing reviews have primarily focused on specific dimensions or contexts of AI within the field of physical education. For example, Zhou et al. conducted a systematic review of AIPE from the perspectives of educators, learners, and administrators, summarizing the overall status, algorithmic applications, and pedagogical impacts (1). However, their discussion of ethical risks was confined to a few general observations and did not offer a systematic categorization. Similarly, Zhong et al. reviewed digital-intelligence technologies in physical education and mentioned ethical risks such as algorithmic accuracy and data privacy (7), yet their analysis remained largely descriptive and did not extend to mitigation strategies. Lai employed a SWOT analysis framework to scope ChatGPT’s role in physical education and sports, highlighting risks including academic integrity, algorithmic bias, data privacy, and homogenized teaching (10), but fell short of proposing systematic alleviation pathways. Although ethical discussions regarding AIPE are becoming increasingly extensive, existing research largely consists of fragmented dimensional analyses and has yet to establish a holistic review framework capable of systematically identifying, categorizing, and addressing ethical risks at various levels. Building upon the classification of AIPE-related ethical risks, this systematic review further distills appropriate alleviation strategies. This study offers dual value in terms of both theoretical deepening and practical guidance. At the theoretical level, it helps to clarify the conceptual understanding of AIPE ethical risks, revealing the specific manifestations of different risks and their intrinsic connections, thereby providing a more refined and comprehensive analytical perspective. At the practical level, it offers clear insights and actionable solutions to diverse stakeholders, including physical education practitioners, learners and AI developers, thereby promoting the development of a responsible, trustworthy and ethical application ecosystem for AIPE. The research questions of this study are as follows:

- In the field of AIPE, what are the typical ethical risks, their causes, and the resulting harms?
- How can the identified ethical risks in AIPE be systematically alleviated?

The remainder of this paper is organized as follows. The methods section describes our protocol and registration, search strategy, inclusion and exclusion criteria, study selection, quality assessment, and coding methods; the results section presents the distribution of characteristics across the included studies and systematically displays the categorization of AIPE ethical risks; and the discussion section traces the causes and interrelationships of various risks and then distills targeted alleviation strategies from three dimensions: technology, physical education, and the body. Finally, the conclusion section provides an overall summary of this study, acknowledges its limitations, and identifies directions for future research.

## 2 Methods

### 2.1 Protocol and registration

This systematic review followed the Preferred Reporting Items for Systematic Reviews and Meta-Analyses (PRISMA 2020) guidelines (11) and was registered in PROSPERO (International Prospective Register of Systematic Reviews) under the registration number-CRD420261338255.

### 2.2 Search strategy

A structured Boolean search strategy was employed to systematically search six electronic databases, including SpringerLink, Web of Science, EBSCOhost, ScienceDirect, Scopus and CNKI.

The search strategy was developed around three core concept clusters: (a) artificial intelligence, including terms such as “artificial intelligence,” “machine learning,” “deep learning,” “ChatGPT,” and “Generative AI”; (b) physical education, including “physical education,” “school physical education,” and “physical education teaching”; and (c) ethics, including “ethic*,” “ethical risk*,” “ethical issue*,” “ethical challenge*,” “privacy,” “algorithmic bias,” “fairness,” “transparency,” and “accountability.” The search syntax was adapted to the indexing rules and search functionalities of each database while maintaining conceptual consistency across all platforms.

To maximize search sensitivity while maintaining specificity and minimizing irrelevant retrievals, synonymous and related terms within each concept cluster were combined using the Boolean operator OR, whereas the three concept clusters were linked using AND to ensure that retrieved records simultaneously addressed artificial intelligence, physical education, and ethical issues. Where supported by the database, searches were restricted to the Title, Abstract, and Keywords fields to improve retrieval precision and reduce irrelevant records. The complete search strategies for all databases are provided in Supplementary material 1.

To further minimize the risk of missing eligible studies, backward and forward citation tracking was performed using Google Scholar. All retrieved records were imported into EndNote 2025 (Clarivate Analytics) for both automatic and manual duplicate removal before the study selection process.

### 2.3 Inclusion and exclusion criteria

The eligibility criteria were established using the SPIDER framework (Sample, Phenomenon of Interest, Design, Evaluation, and Research type), which ensured that study selection was aligned with the review objective and appropriate for synthesizing heterogeneous evidence on ethical issues associated with AIPE. For Sample (S), eligible studies involved stakeholders directly related to physical education, such as students, physical education teachers, educational administrators, and other relevant stakeholders, within formal physical education settings. Studies conducted exclusively in professional or competitive sport, recreational sport, clinical rehabilitation, general fitness, community fitness, or healthcare settings were excluded unless they had an explicit connection to physical education. For the Phenomenon of Interest (PI), studies were required to examine the AI applications, implementations, uses, or implications in physical education with identifiable ethical issues. Studies solely on AI technical development, algorithm optimization, system performance, or teaching effectiveness were excluded if they had no identifiable ethical implications for physical education.

Regarding Design (D), original empirical studies employing an identifiable research design that generated evidence relevant to ethical issues in AI-supported physical education were eligible, including surveys, interviews, focus groups, observations, case studies, experimental studies, cross-sectional studies, longitudinal studies, and mixed-methods designs. For Evaluation (E), studies were required to identify, examine, report, or substantively discuss ethical risks or concerns, adverse consequences, underlying causes, stakeholder impacts, or mitigation and governance strategies associated with AI in physical education. Studies that addressed only technological effectiveness, prediction accuracy, learning or physical fitness outcomes, usability, adoption intention, or teaching efficiency, without identifiable ethical implications, were excluded. For Research type (R), peer-reviewed qualitative, quantitative, and mixed-methods original research articles published in English or Chinese were eligible. Reviews, meta-analyses, bibliometric studies, editorials, commentaries, letters, conference abstracts or papers, book chapters, dissertations, protocols, technical reports, white papers, and other non-original or non-peer-reviewed publications were excluded.

In addition, studies were required to have been published between January 2016 and May 2026, to be available in full text, and to provide sufficient information to establish a substantive relationship among AI, physical education, and ethical issues. The detailed inclusion and exclusion criteria according to each SPIDER component are presented in Table 1.

**Table 1:** Inclusion and exclusion criteria for this systematic review.

Spider element	Inclusion criteria	Exclusion criteria
Sample	Studies involving stakeholders directly related to physical education, including students, physical education teachers, educational administrators, parents, technology-related stakeholders, or other stakeholders involved in AIPE within formal educational settings	Studies conducted exclusively in elite/professional sport, competitive sport, recreational sport, clinical rehabilitation, community fitness, general fitness, or healthcare settings without an explicit connection to formal physical education
Phenomenon of Interest	Studies examining the application, implementation, use, or implications of artificial intelligence or AI-enabled technologies in physical education, where ethical issues or ethically relevant implications were identifiable	Studies focusing exclusively on AI technical development, algorithm optimization, system performance, or teaching effectiveness without identifiable ethical issues or implications related to physical education
Design	Studies employing identifiable research designs, such as interviews, focus groups, surveys, observations, case studies, or other designs capable of generating evidence relevant to ethical issues in AIPE	Studies without an identifiable research design or without analyzable original research evidence
Evaluation	Studies identifying, examining, reporting, or substantively discussing ethical risks, ethical concerns, adverse consequences, underlying causes, stakeholder impacts, or mitigation/governance strategies associated with AIPE	Studies reporting only technological effectiveness, prediction accuracy, learning performance, physical fitness outcomes, usability, adoption intention, or teaching efficiency
Research type	Peer-reviewed qualitative, quantitative, and mixed-methods original research articles published in English or Chinese	Reviews, meta-analyses, bibliometric studies, editorials, commentaries, letters, conference abstracts or papers, book chapters, dissertations, protocols, technical reports, white papers, and other non-original or non-peer-reviewed publications. Articles not published in English or Chinese

### 2.4 Study selection

**Figure 1:**
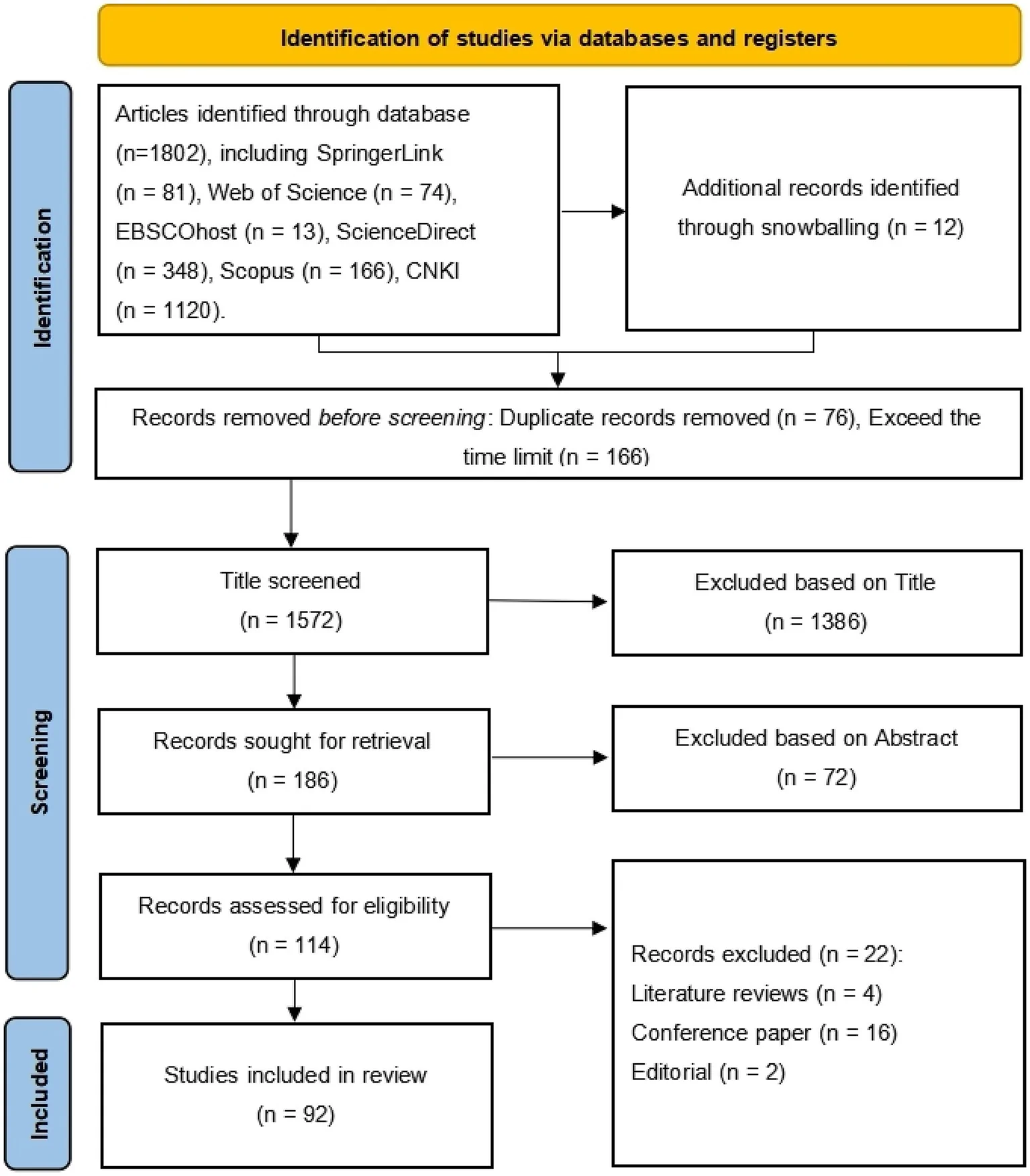
PRISMA flowchart diagram of the study selection process.

The study employed a structured five-stage screening process. (a) Deduplication and temporal filtering: Duplicate records and studies outside the predefined timeframe were excluded. (b) Title screening: Irrelevant studies were removed based on title assessment. (c) Abstract screening: Abstracts were assessed against the eligibility criteria. (d) Full-text review: Two reviewers (Shun Chen and Chaojun Zhang) independently assessed the remaining full-text articles for eligibility. (e) Consensus resolution: Any disagreements were resolved through discussion until consensus was reached. A total of 1,802 records were identified through systematic searches of six electronic databases: SpringerLink, Web of Science, EBSCOhost, ScienceDirect, Scopus and CNKI. In addition, 12 records were identified through backward and forward citation tracking using Google Scholar. After removing 76 duplicate records and 166 records published outside the predefined time frame, 1,572 records remained for title and abstract screening. Following title and abstract screening, 1,458 records were excluded for failing to meet the predefined eligibility criteria, leaving 114 articles for full-text assessment. During the full-text review, 22 articles were excluded. Ultimately, 92 articles (Supplementary material 2) met the eligibility criteria and were included in the final analysis of this systematic review. Screening decisions at each stage based on the SPIDER elements are presented in Supplementary material 3. Reasons for exclusion at the full-text stage are reported in Supplementary Table S1. The overall study selection process is illustrated in the PRISMA flowchart diagram (Figure 1).

### 2.5 Data extraction

This study strictly adhered to the predefined inclusion and exclusion criteria, search strategy, and screening procedures. One researcher (Shun Chen) independently extracted the data using a standardized Excel spreadsheet, and a second researcher (Chaojun Zhang) subsequently verified the extracted information. Any discrepancies were resolved through discussion until consensus was reached. The primary outcome domain was AI-related ethical risks in physical education, defined as reported ethical concerns, harms, tensions, or adverse implications associated with the development, application, or use of AI in physical education. For each included study, all findings, conclusions, and author interpretations that were explicitly relevant to this outcome domain were sought and extracted, irrespective of the specific measure, analytic approach, or form in which they were reported. Other extracted variables included the study title, authors, year of publication, target population, educational level, study design, AI technologies and applications, and other study characteristics relevant to the synthesis. Where information was missing or insufficiently reported, it was recorded as “not reported” or “unclear,” as appropriate; no missing information was imputed or inferred beyond what was explicitly reported in the original study.

### 2.6 Quality assessment

This study employed the Mixed Methods Appraisal Tool (MMAT, version 2018) (12, 13) to assess the methodological quality of the included studies. The MMAT (Supplementary material 4) provides design-specific criteria for five study categories: qualitative studies, randomized controlled trials, non-randomized studies, quantitative descriptive studies, and mixed methods studies. Following the MMAT user guide, each study was assessed against the criteria corresponding to its design. Two reviewers (Shun Chen and Chaojun Zhang) independently conducted the quality appraisal, and disagreements were resolved through discussion, with a third reviewer consulted when necessary. The MMAT does not recommend deriving an overall score from individual criterion ratings; instead, it emphasizes reporting the appraisal results for each criterion to provide a more comprehensive representation of the methodological strengths and limitations of the included studies. Detailed appraisal results are presented in Supplementary material 5.

### 2.7 Coding

This study employed a mixed deductive-inductive thematic analysis. The analytical procedures were informed by the hybrid thematic analysis proposed by Fereday and Muir-Cochrane (14) and followed the thematic analysis framework developed by Braun and Clarke (15, 16). During the deductive phase, the study drew on the ethical risk categories and organizational framework synthesized by Zhu et al. (17). Their technology, education, and society dimensions, together with the associated risk categories, served as sensitizing concepts and sources for the initial codes. These categories were then contextually adapted to reflect the distinctive characteristics of physical education, including physical activity, motor skill learning, performance assessment, teacher-student interaction, and bodily development, resulting in a preliminary *a priori* coding framework. During the inductive phase, the researchers remained open to concepts emerging from the included studies. When an ethical risk could not be adequately explained or accommodated within the existing framework, new codes were generated from the findings and discussions reported in the original studies. The coding framework was progressively refined through constant comparison, merging, splitting, and revision. In particular, risks related to bodily experience, bodily autonomy, body boundary, and body value were not predetermined as fixed categories but were identified and constructed primarily through inductive analysis. The final themes and their hierarchical structure were therefore jointly shaped by the theoretical framework and the reviewed evidence rather than derived through the direct application of an existing classification system.

Two coders (Shun Chen and Chaojun Zhang) independently coded all included studies in NVivo 15. The coding process was divided into two stages. The first stage was precoding and calibration, in which the two researchers independently conducted two rounds of precoding on all included studies. By comparing the results, we improved the coding manual, standardized operating procedures, and reached a consensus on coding definitions. The second stage was formal coding, in which the two researchers performed a third round of coding on all studies based on the finalized manual. The coding unit was single research finding, conclusion, or author interpretation related to the ethical risks of artificial intelligence in physical education. For semantic units belonging to multiple categories, multiple codes were permitted. Inter-coder agreement was calculated using Holsti’s Coefficient (18, 19). The formula is: *2a*/(*2a + b + c*), where *a* represents the number of units on which both coders agreed, *b* denotes the number of units coded exclusively by the first coder, and *c* denotes the number of units coded exclusively by the second coder. The resulting coefficient was 0.898, indicating a substantial level of agreement. Inter-coder disagreements were resolved through discussion, and a fourth round of coding was completed with full consensus. An audit trail of coding decisions, codebook revisions, and NVivo records ensured analytical transparency and traceability. An illustrative example of the coding framework for ethical risks in the physical education dimension is presented in Table 2. The complete coding framework is available in Supplementary material 6. All original text data have been stored and managed in the NVivo software system.

**Table 2:** Basic information on coding system (exemplifying the physical education dimension).

Main categories	Preliminary categories	Concepts	Original statements
Physical education	Physical education teaching	Homogeneous physical education teaching	Although AI-driven personalized instruction aims to accommodate individual differences, it may evolve into a constrained form of standardization driven by technological logic, thereby limiting its flexibility in addressing students’ diverse needs (74).
		Threats to teachers’ professional roles and agency	Otherwise, the high ability level offered by an AIPE teacher may result in PE teaching as a human profession disappear (79).
		Deviation from the goals of physical education	AI has an inherent tendency to reinforce the logic that “what is measurable is valuable,” thereby contributing to the alienation of educational goals and the displacement of the value-oriented functions of physical education to some extent (74). While the platform enhances efficiency and personalization, it risks reducing the social and emotional benefits of traditional PE, such as peer collaboration and instructor-student rapport (20).
	Physical education learning	Homogenization of student development	Market-driven intelligent physical education programs tend toward standardized, replicable teaching templates. This homogenized temporal object erases the individual differences of students in physical education, and mistakenly casts students into the image of abstract, homogenized “consumers” (22).
	Teacher-student relationship	Alienation in teacher-student relationships	Most (77.78%) teachers believed that artificial intelligence would have a greater impact on the relationship between physical education teachers and students in the future (81).
		Alienation in student–student relationships	AI may hinder authentic interactions among students and between students and real-world contexts (86). Scepticism revolved around AI’s real-world value and fears that over-reliance on technology could undermine human interaction and skill development (100).
	Humanistic value	Lack of humanistic care	The design of AI-enabled systems may prioritize the quantification of health, physical fitness, and motor skills while overlooking or diminishing the moral and humanistic dimensions of physical education (81).
		Value alienation	AI technologies rely heavily on programmed algorithms and training data, making their outputs susceptible to value-laden biases embedded in the data, such as those related to gender, culture, and race. These biases may, in turn, subtly shape the values of learners engaged in self-directed learning (106).
	Academic research	Academic misconduct	When students use DeepSeek to generate personalized course papers or assignments, the absence of effective content detection mechanisms may also give rise to concerns regarding academic integrity (38).

This methodology aimed to identify and classify the ethical risks associated with AIPE systematically and to explore their causes and hazards.

## 3 Results

### 3.1 Study characteristics

#### 3.1.1 Methodological composition of the included studies

A total of 92 studies were included in this systematic review. The list of included studies and their quality assessment results are provided in Supplementary materials 2, 5, respectively. Regarding the methodological composition, 74 were qualitative studies, 1 was a randomized controlled trial, 8 were non-randomized studies, 3 were quantitative descriptive studies, and 6 were mixed-methods studies. The thematic analysis in this review was primarily grounded in the contextual and experiential evidence derived from the qualitative studies, which provided rich insights into the ethical risks of AIPE. Meanwhile, the limited number of quantitative and mixed-methods studies offered complementary empirical evidence regarding the scale and domain-specific occurrence of certain risks. Collectively, the integration of evidence across these different study designs provided the empirical foundation for the thematic categories and overall framework developed in this review.

#### 3.1.2 Distinct AI applications and associated ethical concerns

As detailed in Supplementary Table S2, four primary categories of AI applications in physical education were identified among the included studies, each associated with distinct ethical concerns. The findings indicate that the emphasis of ethical risks varies according to the type of AI application. (a) Generative AI tools (e.g., ChatGPT, DeepSeek) predominantly raised issues concerning reliability, authorship, academic integrity, and inappropriate dependence. (b) AI-enabled monitoring and wearable systems primarily foregrounded privacy, continuous surveillance, informed consent, and data security. (c) Computer-vision or movement-analysis systems were mainly associated with concerns regarding measurement accuracy, model limitations, and algorithmic bias across different body types or movement patterns. (d) AI-supported assessment, recommendation, or learning-analytics systems were particularly implicated in transparency, fairness, standardization, and pedagogical autonomy.

#### 3.1.3 Distribution of journals

The 92 included studies were published across 47 journals, indicating a relatively broad but uneven journal distribution. The Journal of Shenyang Sport University contributed the largest number of articles (*n* = 13), followed by the Journal of Sports Research and Sports Culture Guide (*n* = 6 each) and China Sport Science (*n* = 5). The Journal of Physical Education and Journal of Shanghai University of Sport each published four articles, while five journals contributed three articles each. The Journal of Guangzhou Sport University, Frontiers in Public Health, and Scientific Reports each contributed two articles. The remaining 33 journals each published one article and were grouped as “Other journals” in Table 3. The complete journal distribution is presented in Supplementary material 7. The above results indicate that most of the included studies were published in authoritative Chinese journals.

**Table 3:** Number of included studies (*n* = 92) by journal.

Rank	Journal	*n*
1	Journal of Shenyang Sport University	13
2	Journal of Sports Research	6
3	Sports Culture Guide	6
4	China Sport Science	5
5	Journal of Physical Education	4
6	Journal of Shanghai University of Sport	4
7	The Journal of Physical Education, Recreation & Dance	3
8	Journal of Xi’an Physical Education University	3
9	Journal of Beijing Sport University	3
10	Journal of Chengdu Sport University	3
11	Sports & Science	3
12	Journal of Guangzhou Sport University	2
13	Frontiers in Public Health	2
14	Scientific Reports	2
15–47	Other journals (33 journals; one article each)	33

#### 3.1.4 Distribution of years

**Figure 2:**
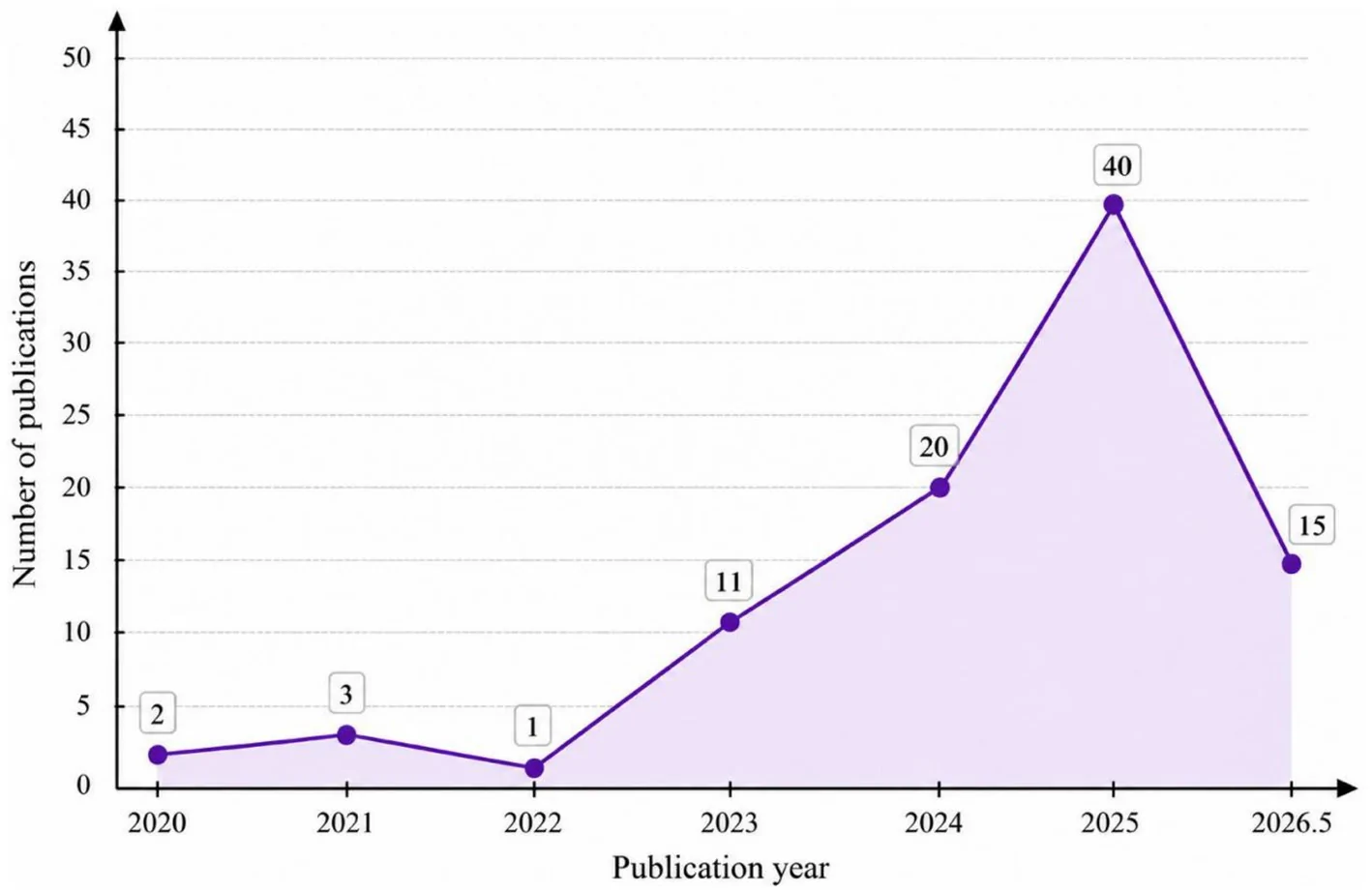
Number of included studies per year (*n* = 92).

The number of the 92 included studies per year showed an overall upward trend over time (Figure 2). Publication output peaked in 2025, when 40 studies were published.

### 3.2 Risk of bias in studies

Quality assessment results are presented in Supplementary material 5. Overall, methodological quality varied across study designs. All 92 studies met the two MMAT screening criteria. Among the 74 qualitative studies, all met the criterion concerning the appropriateness of the qualitative approach, and 69 adequately addressed the research question through appropriate data collection methods. However, greater variability was observed in the derivation of findings from the data, substantiation of interpretations, and coherence between data sources, collection, analysis, and interpretation. The single randomized controlled trial met the criteria for randomization and baseline comparability but showed limitations regarding completeness of outcome data, blinding, and adherence to the assigned intervention. Among the eight non-randomized studies, methodological limitations were mainly related to participant representativeness and the consideration of confounding factors. The three quantitative descriptive studies generally met criteria concerning sampling strategy, measurement, and statistical analysis, although some concerns remained regarding sample representativeness and non-response bias. All six mixed methods studies met the criteria related to the rationale, integration, interpretation, and management of divergences between qualitative and quantitative components; however, only one fully met the quality criteria for each component of the mixed methods design. Consistent with MMAT guidance, no overall numerical quality score was calculated, and studies were not excluded solely based on methodological quality. All studies were therefore included for analysis.

### 3.3 Coding results

**Figure 3:**
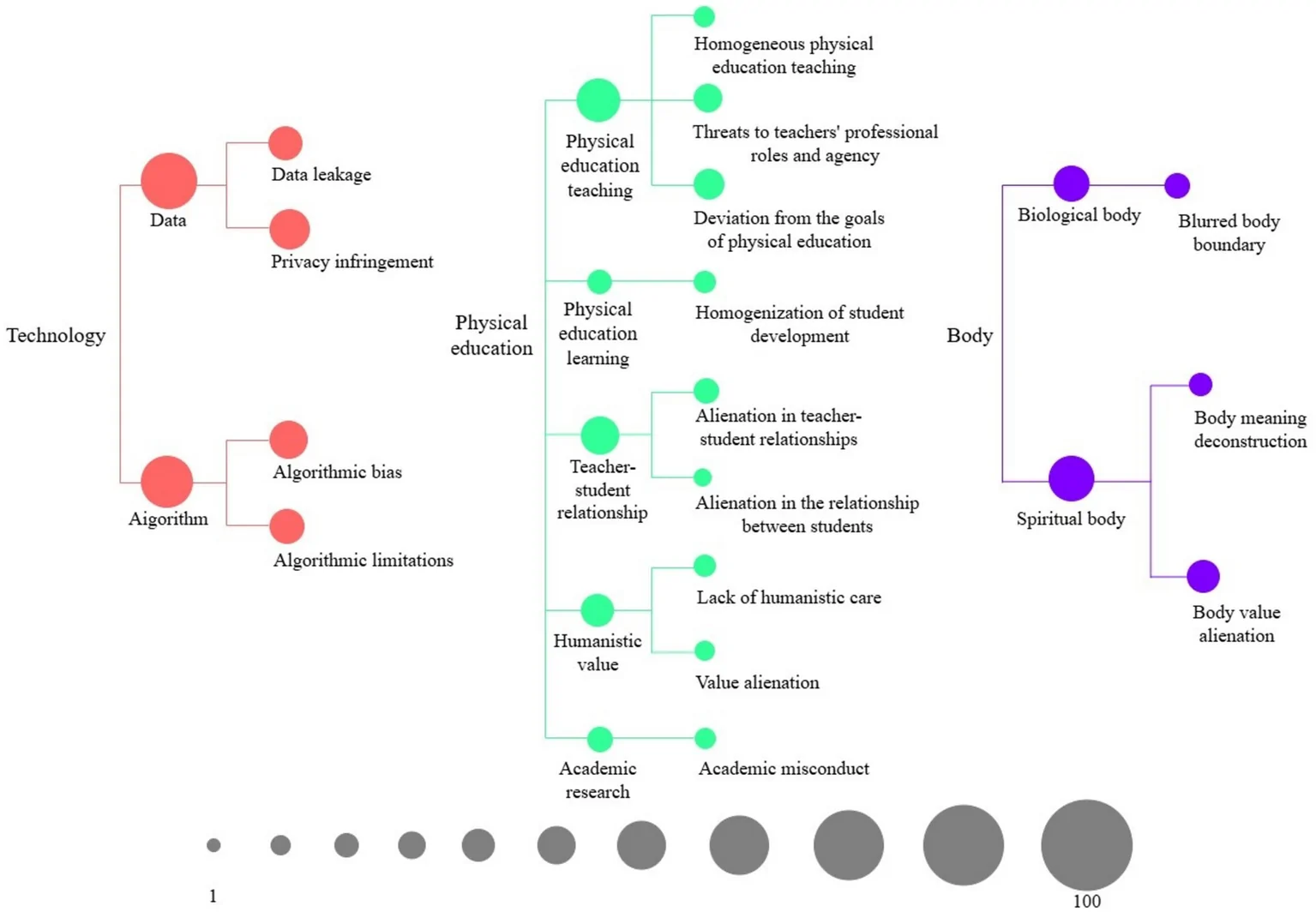
The frequency of the ethical risks of AIPE.

The coding results were used to quantify the frequency of ethical risks identified in the included studies, as presented in Figure 3.

#### 3.3.1 The ethical risks of AIPE from the technology dimension

The technology dimension encompasses the ethical risks associated with AIPE. At the data level, these risks include data leakage and privacy infringement. At the algorithmic level, they comprise algorithmic bias and algorithmic limitations.

*Data leakage.* Among the 92 included studies, 53 identified this risk. AI continuously collects data such as heart rate, movement trajectories, movement patterns, and learning behaviors, supporting personalized instruction while increasing sensitive data exposure (20, 21). Leakage may violate privacy, undermine trust, and enable data misuse, unauthorized profiling, algorithmic discrimination, and discriminatory pricing without informed consent (22–25). The factors contributing to data leakage are as follows. (a) Data security vulnerabilities: system flaws, inadequate encryption, weak access controls, cyberattacks, centralized storage, and cross-platform transmission increase unauthorized data disclosure, theft, and manipulation (21, 26, 27). (b) Inadequate data governance: unclear ownership, weak institutional safeguards, excessive data collection and sharing, commercial exploitation, and regulatory gaps heighten leakage risks (28–30).

*Privacy infringement.* Among the 92 included studies, 64 identified this risk. Wearable devices, computer vision, biosensors, and learning analytics continuously collect bodily data, such as heart rate, movement trajectories, and performance, increasing privacy risks (20, 31). Continuous surveillance may also create psychological pressure, feelings of being monitored, and fairness concerns (32). Privacy infringement can weaken trust and technology acceptance while reducing students to data objects vulnerable to distress, labeling, and discrimination (22, 25, 33). Several factors contribute to the emergence of privacy infringement, as outlined below. (a) Inadequate system security protection: weak security-by-design, network vulnerabilities, and limited safeguards expose sensitive data to unauthorized access, theft, and misuse (22, 26, 27). (b) Failure of informed consent: unobtrusive biometric and behavioral data collection may render consent ineffective and subject students to persistent algorithmic monitoring (20, 31, 33).

*Algorithmic bias.* Among the 92 included studies, 59 coded excerpts identified algorithmic bias as an ethical risk. Algorithmic bias not only undermines fairness in sports performance assessment, resource allocation, and learning support but may also reinforce historical biases, cultural stereotypes, and group labeling, suppressing diversity in students’ physical characteristics, cultural backgrounds, and learning needs and ultimately diminishing the humanistic orientation and equity of physical education (34–37). The causes of algorithmic bias can be broadly categorized into three aspects. (a) Data bias. Training datasets may suffer from insufficient sample size, limited representativeness, historical bias, or labeling bias, causing AI models to inherit or even amplify existing biases during training and consequently produce systematic errors in algorithmic outputs (21, 23, 24, 38–40). (b) Algorithmic model bias. Unreasonable assumptions, black-box mechanisms, and the predictive limitations of algorithmic models reduce the transparency of decision-making and may further exacerbate digital discrimination, structural inequalities in educational opportunities, and imbalances in assessment (32, 35, 41, 42). (c) Application bias. AI-generated content is jointly influenced by algorithmic filtering mechanisms, data quality, and contextual suitability. In complex physical education settings, this may result in factual inaccuracies, insufficient cultural sensitivity, and inadequate adaptation to individual needs (36, 43–47).

*Algorithmic limitations.* Among the 92 included studies, 55 identified this risk. AI lacks consciousness, emotion, and reflective thinking (48–50), making it poorly aligned with the embodied experiences, perceptions, emotions, and interactions central to physical education (51). It cannot fully reproduce human creativity (52) or replace PE teachers’ higher-order pedagogical reasoning and emotional engagement (53–57). Algorithms also struggle to capture unstructured elements such as humanistic values, passion, and respect (41, 58–63), potentially generating unreliable information that compromises teaching decisions and students’ physical health (36, 39, 64). Standardized data logic may overlook bodily differences, educational contexts, and cultural backgrounds, producing evaluations that fail to reflect actual needs (47, 65). Large language models may also generate factual errors or hallucinations, particularly when addressing specialized terminology and complex movements, undermining teaching and research credibility (43–45, 66–68). The causes contributing to algorithmic limitations are outlined as follows. (a) Data and model limitations: Incomplete, low-quality, or insufficiently diverse data and algorithm-design constraints reduce output accuracy and stability (21, 39, 64). (b) Inadequate adaptation to complex physical education contexts: Algorithms inadequately account for bodily differences, teaching situations, and cultural settings, creating discrepancies between AI outputs and educational needs (20, 47, 65). (c) Algorithmic opacity: Non-transparent decision-making and recommendation mechanisms make algorithmic outputs difficult for teachers and students to interpret (69–73).

#### 3.3.2 The ethical risks of AIPE from the physical education dimension

Results indicate that ethical risks within the dimension of physical education manifest primarily in five areas: physical education teaching, physical education learning, teacher-student relationships, humanistic value, and academic research. At the physical education teaching level, these include homogeneous physical education teaching, threats to teachers’ professional roles and agency, and deviation from the goals of physical education. At the physical education learning level, they involve risks related to homogenization of student development. Regarding the teacher-student relationship, ethical risks associated with emotional conflicts include alienation in teacher-student relationships and alienation in student–student relationships. The humanistic value dimension manifests in a lack of humanistic care and value alienation. The academic research dimension, meanwhile, is characterized by academic misconduct.

*Homogeneous physical education teaching.* The coding identified 19 excerpts concerning this risk. Standardized AI-generated content, demonstrations, and assessments may improve efficiency but produce “limited standardization” in personalized instruction (22, 47, 74). Uniform movement standards may encourage mechanical imitation, restrict exploration and creativity, and increase injury risks (21, 54). Dependence on standardized lesson plans may also shift PE teachers from educational decision-makers to technological executors, leading to routinized teaching, weakened agency, and burnout (35, 58, 75). The following factors were identified as contributing to homogeneous physical education teaching. (a) The inertia of traditional physical education teaching: skill-oriented methods, outdated beliefs, and limited digital literacy perpetuate standardized instruction (26, 38, 76). (b) Alienation of technological intervention: market-driven standardization and algorithmic reinforcement promote templated teaching and weaken teacher and student creativity (22, 75). (c) Constraints on resource allocation. Fragmented digital resources and siloed platforms increase the costs of resource integration, prompting physical education teachers to rely on standardized lesson plans and further exacerbating homogeneous physical education teaching (77).

*Threats to teachers’ professional roles and agency.* The coding identified 26 excerpts concerning this risk. As AI assumes teaching design and classroom assessment, teachers’ professional judgment and authority may erode, reducing them from “lead instructors” to executors of technological procedures (35, 59). Procedural digital management may also weaken interpersonal relationships, dialogue, ethical care, and teachers’ educational agency (44, 78). Advanced AI may potentially replace PE teachers (79, 80), while replacement concerns may generate career anxiety, psychological stress, and “quiet quitting” (81–83). These threats may weaken professional identity and career motivation, reduce willingness to enter or remain in the profession, and worsen workforce imbalances and shortages of high-quality teachers (23, 80). The reasons contributing to threats to teachers’ professional roles and agency are delineated as follows. (a) Substitutive threat of artificial intelligence: AI narrows teachers’ professional boundaries and challenges the value of their physical presence (65, 83–85). (b) Insufficient digital literacy among PE teachers: Limited digital competence increases pressure to transform professional roles (29, 49).

*Deviation from the goals of physical education.* The coding identified 42 excerpts concerning this risk. Technology-driven instrumental rationality may marginalize the humanistic aims of physical education, shifting attention from holistic development toward measurable outcomes, technological optimization, and data performance (41, 61, 65, 74). This assessment logic may encourage students and teachers to prioritize movement standards, attainment rates, and scores while neglecting emotions, individuality, critical thinking, and humanistic care, transforming physical education from “educating the whole person” into “educating for scores” and “educating for data” (32, 37, 55, 65, 77). Deviation from the goals of physical education was associated with three principal factors. (a) Technological overreach: Efficiency-oriented logic privileges measurable objectives and compresses space for humanistic education and emotional development (61, 65, 74). (b) Algorithmic limitations: AI struggles to identify implicit goals such as emotional experience and value formation, potentially misaligning resources with students’ developmental needs (37, 56, 86). (c) PE teacher role failure: Excessive reliance on AI-generated content may obscure individual differences and allow AI to shift from an auxiliary tool to a source of value orientation (58, 59, 83).

*Homogenization of student development.* Among the included studies, 30 coded excerpts referred to homogenized student development. Algorithmic “personalization” does not necessarily promote genuine individual development and may instead produce new forms of homogenization. AI may recommend similar content to students, resulting in convergence in skill acquisition and cognition and limiting its capacity to address diverse developmental needs (21, 37, 47, 65). Because algorithmic recommendations are often generated from group-level average data, they may overlook individual differences and place students within a uniform technological framework (60, 68). Excessive reliance on AI may also weaken students’ autonomous learning and inhibit their agency and creativity (39, 55, 66, 84). It may further undermine cooperation, communication, leadership, and social interaction, fostering an instrumental orientation toward learning and hindering students’ holistic development (39, 80). Homogenization of student development primarily arises from the following factors. (a) Technological dependence: prolonged reliance on AI for learning guidance, assessment feedback, and learning plans may weaken students’ independent judgment and critical thinking, thereby reducing opportunities for independent exploration and creative development (55, 71, 84). (b) Information cocoons: personalized recommendations may create information cocoons that restrict students’ exposure to diverse knowledge, differing perspectives, and heterogeneous experiences, narrowing their learning horizons and reinforcing homogenizing tendencies (39, 58).

*Alienation in teacher-student relationships*. The coding identified 46 excerpts concerning this risk. AI has transformed the traditional “teacher-student” structure into a “teacher-AI-student” structure, reducing face-to-face communication and weakening embodied experience, immediate feedback, and empathy (44, 49, 55, 65). Students may increasingly seek support from AI rather than teachers, diminishing teachers’ presence and epistemic authority while hindering communication, cooperation, and empathy (27, 83, 87). The primary factors contributing to alienation in teacher-student relationships are as follows. (a) Technological mediation replaces direct interaction: reliance on intelligent technologies shifts teacher-student interaction toward human-machine interaction, weakening emotional connection and humanistic care (28, 49, 55, 77, 83, 84). (b) Instrumental rationality constrains relational values: AI prioritizes efficiency, precision, and data management while marginalizing relationship building, value guidance, and emotional development (35, 74, 85).

*Alienation in student–student relationships.* The coding identified 28 excerpts concerning this risk. Technological mediation may weaken peer relationships based on bodily co-presence, cooperative competition, and emotional communication, reducing the role of physical education as a community of social learning (27, 44, 84). Excessive reliance on AI feedback may reduce authentic interaction, weaken collective identity and belonging, and hinder socio-emotional development (55, 74, 75, 80, 87). The reason for alienation in student–student relationships is as follows. Technological mediation replaces authentic peer interaction: although virtual platforms and intelligent feedback may improve efficiency, they reduce face-to-face communication, collaborative inquiry, and bodily interaction (28, 54, 55). Prolonged technological immersion may foster dependence, emotional detachment, and social isolation, thereby weakening communication, cooperation, shared exploration, and the social-development function of physical education (49, 52, 85).

*Lack of humanistic care.* The coding identified 32 excerpts concerning a lack of humanistic care. As AI becomes widely used in physical education, teaching increasingly emphasizes movement standards, skill attainment, and data feedback, while giving less attention to students’ emotions, cooperation, willpower, and value development (22, 77). Prolonged exposure to technology-dominated environments may weaken emotional connections between teachers and students and among students, making classroom interaction more instrumental and undermining the humanistic, moral, and socio-emotional functions of physical education (54, 85). Lack of humanistic care can be primarily attributed to the following factors. (a) Dominance of technological instrumental rationality: AI prioritizes efficiency, precision, and quantitative assessment but lacks humanistic care, limiting emotional and value education (38, 74). (b) PE teacher role failure: dependence on intelligent systems may weaken teachers’ roles in emotional support, value guidance, and character development (49, 58). (c) Deviation from physical education goals: the growing emphasis on quantitative indicators may marginalize humanistic literacy and life education (61, 65).

*Value alienation.* The coding identified 27 excerpts concerning this risk. Prolonged exposure to algorithmic recommendations and AI-generated content may foster technological supremacy, performance orientation, and utilitarian values while weakening students’ identification with sportsmanship, collectivism, humanistic care, and local sports culture (35, 85, 88). The major factors contributing to the occurrence of value alienation are as follows. (a) Deviation from physical education goals: AI prioritizes efficiency, performance, and quantitative outcomes, marginalizing sportsmanship, moral education, humanistic literacy, and value guidance (69, 74). (b) Algorithmic value bias: cultural and corpus biases in training data may distort sports knowledge, recommendations, and cultural communication, weakening local sports culture and appropriate value guidance (38, 45). (c) Weakening of autonomous value judgment: dependence on AI may reduce independent thinking, value discernment, and educational judgment, making decisions increasingly algorithm-driven rather than context-sensitive (71, 88).

*Academic misconduct.* The coding identified 31 excerpts concerning academic misconduct. Generative AI can rapidly produce text, images, and data analyses, making misconduct more concealed, complex, and sometimes unintentional (38, 55). Students may use AI to complete assignments or papers, while teachers may adopt AI-generated teaching or research materials without verification or disclosure, undermining originality, credibility, and academic integrity (38, 44, 55). The emergence of academic misconduct can be explained by the following factors. (a) Inadequate academic governance: unclear standards for AI-assisted writing, authorship, and disclosure, combined with limited digital literacy and awareness of intellectual property and data ethics, increase the risk of concealed academic misconduct (29, 30, 66, 68). (b) Academic pressure and utilitarian orientation under pressure from course assessment, academic publication, and research performance evaluation, some teachers and students use AI as a substitute for thinking, writing, and research design rather than as an auxiliary tool (87, 89).

#### 3.3.3 The ethical risks of AIPE from the body dimension

The body dimension examines the ethical risks of AIPE. At the biological body level, these risks are primarily manifested in blurred body boundary. At the spiritual body level, they are mainly reflected in body meaning deconstruction and body value alienation.

*Blurred body boundary.* The coding identified 21 excerpts concerning blurred body boundary. Blurred body boundary refers to the increasing ambiguity in the boundaries between the biological body and technological systems when AI-enabled devices, wearable technologies, virtual environments, or other forms of human–technology integration extend, mediate, or partially substitute bodily perception, action, and interaction. Through wearable devices, virtual reality, and brain-computer interfaces, AI increasingly obscures distinctions between body and technology, reality and virtuality, and the organic and inorganic, creating ethical challenges for students’ bodily cognition, identity, and subjectivity (24, 35, 75). As bodily functions are enhanced, replaced, or reconstructed, students may struggle to determine “Who am I?” and “What constitutes the real body?”, making the boundary between bodily and technological subjects increasingly uncertain (48). Virtual reality, digital humans, and generative AI further expand bodily activity into virtual spaces. Prolonged immersion may weaken students’ bodily perception, spatial adaptability, and real-world movement identity, while fostering “virtual personas” with different values and behavioral patterns (23, 33, 85). As bodily and intelligent technologies converge, bodily subjectivity may be eroded, and human-machine boundaries blurred (48, 75, 88). Blurred body boundary can be attributed to the following factors. (a) Re-embodiment: enhancement technologies blur the natural-artificial body boundary (48, 75). (b) Technological embodiment: wearables participate continuously in perception, decision-making, and feedback, creating body-technology symbiosis (20, 35). (c) Digital embodiment: digital bodies weaken distinctions between physical and virtual embodiment and undermine bodily self-awareness (24, 33, 90).

*Body meaning deconstruction.* The coding identified 43 excerpts concerning body meaning deconstruction. Body meaning deconstruction refers to the reduction or marginalization of students’ lived bodily experience, affect, agency, interpersonal bodily interaction, and subjective meaning when physical education is increasingly mediated through digital representation, quantification, or algorithmic interpretation. Excessive reliance on AI feedback, virtual simulation, and intelligent decision-making may shift students’ attention from bodily perception and authentic experience to movement data and performance indicators, producing “disembodied movement” (35, 54, 78). This may weaken bodily identity and lived experience, generate body anxiety and loss of self-identity, and erode the role of physical education in supporting harmonious physical and psychological development (71, 75). The following factors were identified as contributing to body meaning deconstruction. (a) Objectification of the bodily subject: AI reduces the body to computable indicators and an object of algorithmic optimization, weakening perception, emotion, and life meaning (35, 48, 65, 78). (b) Technological overreach: AI replaces authentic movement, restricts autonomous exploration, and undermines physical education’s purpose of “educating through the body” (27, 33, 51, 60, 88).

*Body value alienation.* The coding identified 52 excerpts concerning body value alienation. Body value alienation refers to the tendency to evaluate and understand the body primarily through measurable performance, fitness, biometric, or behavioral indicators, while marginalizing the subjective, experiential, relational, and humanistic dimensions of bodily value. Digital technologies prioritize standardization and efficiency, while physical education depends on bodily practice and cultural engagement. Consequently, difficult-to-quantify values, including movement enjoyment, embodied experience, character development, and personal growth, may be marginalized (32, 55, 74, 91). Persistent emphasis on performance data may intensify utilitarian attitudes, body anxiety, and psychological pressure, while diverting physical education from holistic development (75, 78). Body value alienation was associated with two main contributing factors. (a) Quantification-oriented evaluation: AI reduces the body to measurable indicators, transforming it into an object of data production and performance management while constraining movement experience, lived experience, and educational value (32, 54, 55). (b) Algorithmic limitations: AI assumes teaching and guidance functions despite lacking bodily experience, emotion, and ethical judgment, weakening life education, humanistic care, and value formation (48, 51, 62, 70, 88).

## 4 Discussion and implications

### 4.1 The potential ethical risks of AIPE

**Figure 4:**
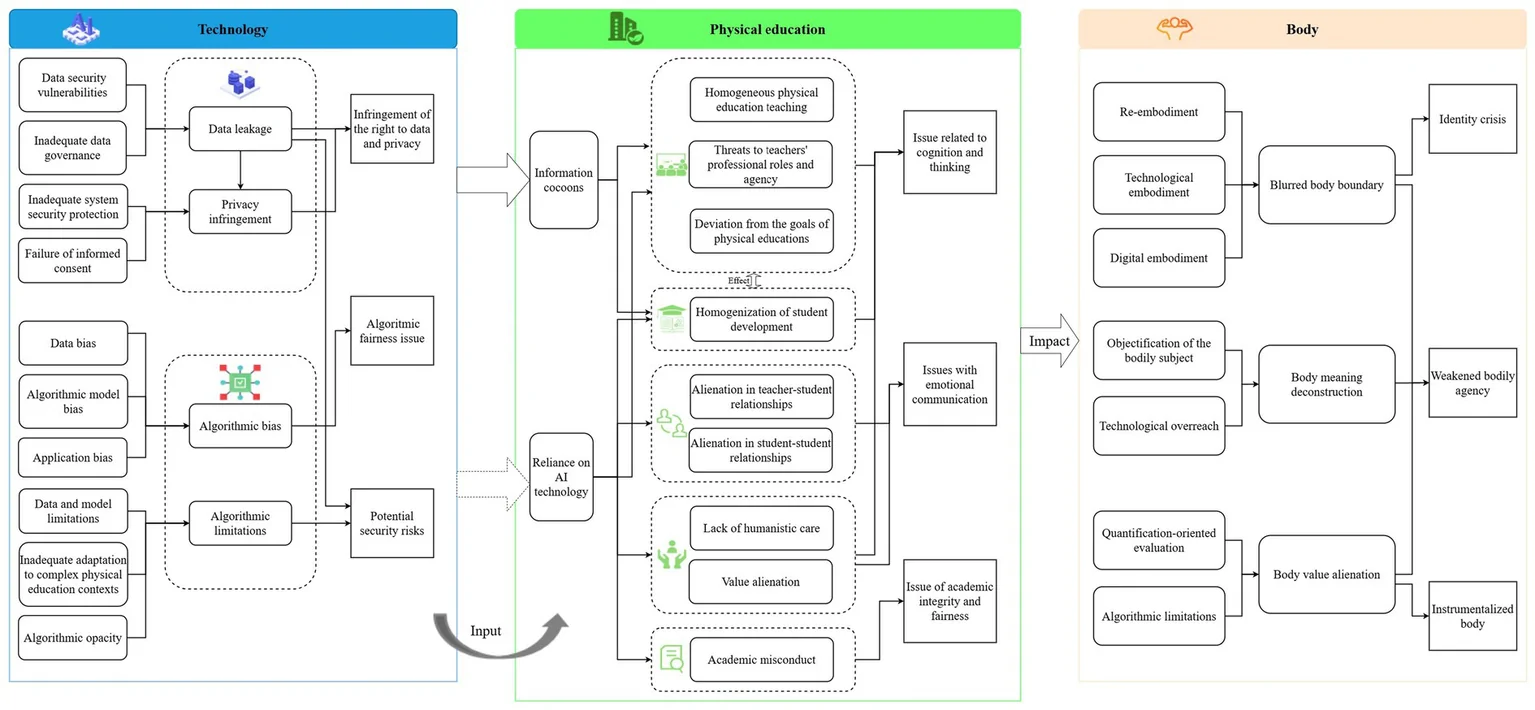
The potential interconnections among the ethical risks of AIPE.

This study found that the three dimensions of ethical risks—the technology dimension, the physical education dimension, and the body dimension—do not exist in isolation but interact in complex ways. Ethical risks arising in one dimension may trigger or exacerbate risks in another, as illustrated in Figure 4.

Technology dimension risks form the upstream layer of the broader risk network, particularly through algorithmic bias, algorithmic limitations, and data leakage. Unrepresentative training data may produce unfair evaluations of students by body type or gender, while data leakage often results from system vulnerabilities and weak data governance. Once embedded in AI-supported systems, these defects extend into physical education practice.

Within physical education, AI technical defects reshape physical education teaching, physical education learning, teacher-student relationships, humanistic value, and academic research. Homogenized algorithmic teaching plans may obscure individual differences, while recommendation systems can create information cocoons that constrain diversity, individuality, and creativity (74). Algorithmic opacity and limited sensitivity to complex pedagogy or emotion may weaken teachers’ autonomy and authority, contributing to a professional crisis. Delegating instructional decisions to technology may also replace direct teacher-student interaction with human-machine communication, intensifying relational alienation and reducing humanistic care (41). Dependence on AI-generated content may further facilitate academic misconduct and deviation from physical education goals (28).

These risks ultimately accumulate in the body dimension. Standardized, repetitive movement may reduce the body from a living subject to a data object, displacing enjoyment, emotion, and volition and contributing to body meaning deconstruction (75). Data-centered evaluation may also instrumentalize the body, obscure its ethical and experiential significance, and produce body value alienation (48). Prolonged immersion in virtual sports may destabilize the distinction between the digital and natural body, disrupt identity formation, and lead to blurred body boundary (24).

### 4.2 Strategies to tackle the potential ethical risks of AIPE

**Figure 5:**
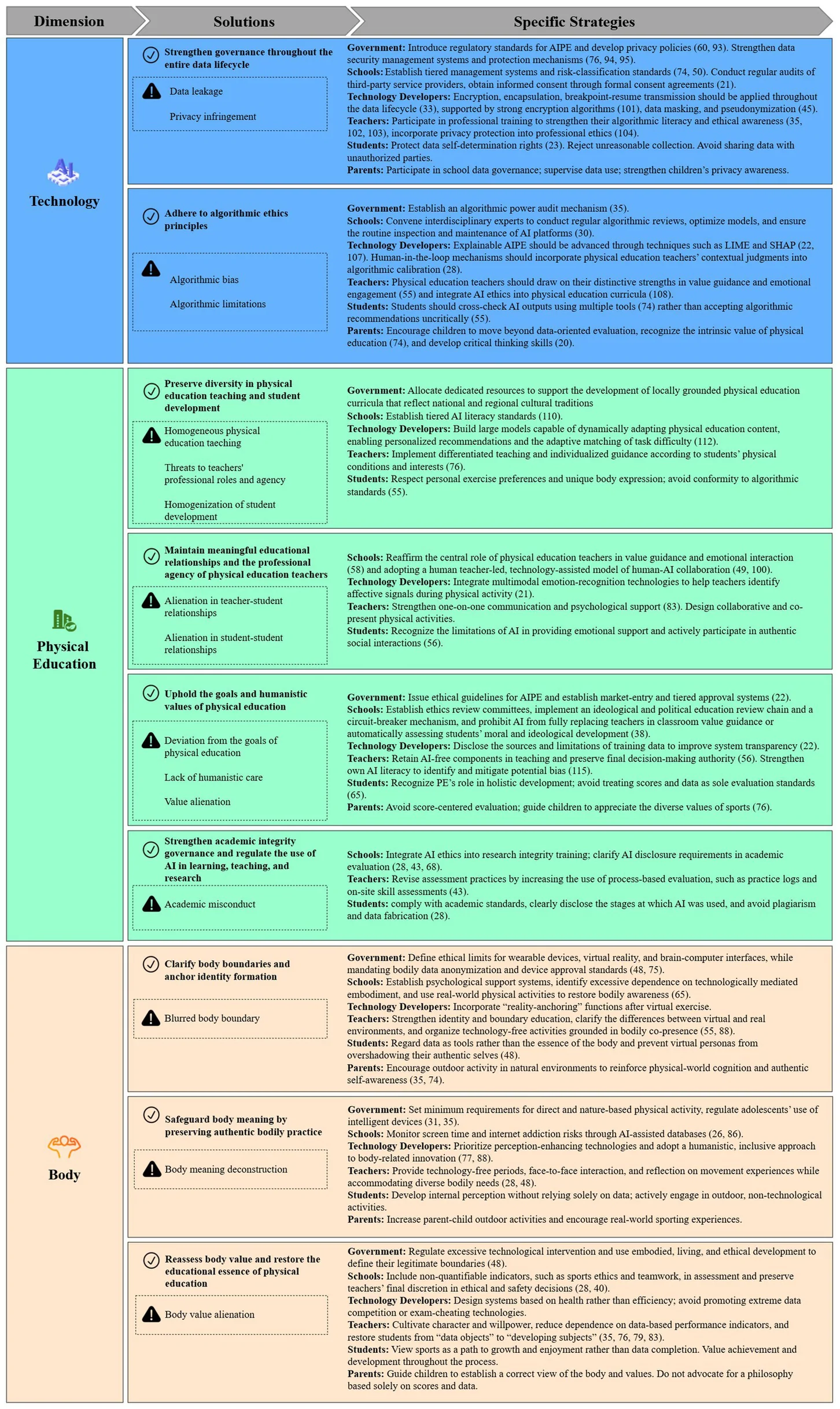
The strategies for alleviating the ethical risks of AIPE.

Based on the systematic identification of AIPE-related ethical risks across 92 studies, this study synthesized targeted risk-alleviation strategies from the perspectives of key stakeholders, including schools, teachers, and students (Figure 5). These strategies provide stakeholder-specific guidance for ethical practice and support the responsible use of AI within different roles and educational contexts. The individual strategies were derived from the included literature, whereas their integration into a coordinated multi-stakeholder framework represents the synthesis conducted in this review. Collectively, they aim to foster a trustworthy AIPE ecosystem with positive feedback and promote responsible, socially beneficial intelligent PE development.

#### 4.2.1 Strategies for the technology dimension

First, establish robust data security and privacy protection systems, strengthen governance throughout the entire data lifecycle. Because ethical risks are embedded throughout the data lifecycle, including data collection, processing, sharing, and reuse (92), comprehensive data lifecycle governance is essential. Governments should introduce regulatory standards for AIPE and develop privacy policies that address both technical requirements and privacy protection (60, 93). They should also strengthen data security management systems and protection mechanisms to ensure the compliant use of AI in physical education teaching (76, 94, 95). Physical education authorities should further define the boundaries of sports and health data collection, data storage permissions, and retention and deletion periods while requiring technology providers to obtain privacy-by-design certification (21). Regulatory frameworks should incorporate the principles of data minimization and revocable authorization, establish emergency response and accountability mechanisms (38). Governance should also coordinate soft-law and hard-law instruments (24) and improve the relevant legal framework (30, 96). Schools should establish tiered management systems and risk-classification standards for physical education data, clearly defining the relevant data subjects and access permissions to ensure that student information is collected and used ethically and prudently (50, 74, 97, 98). They may appoint dedicated data governance officers, strengthen data security education for physical education teachers and students, and increase awareness of data security and privacy protection (96, 99). Schools should also conduct regular audits of third-party service providers, obtain informed consent through formal consent agreements (21), and ensure that teachers and students are adequately informed and that applicable data standards are followed (66, 80, 100). Technology developers should adopt a coordinated cloud-edge-device architecture in which data is de-identified on edge devices and only aggregated features are transmitted to the cloud. Federated learning may further reduce the risks associated with centralized storage of raw data (21). Encryption, encapsulation, breakpoint-resume transmission, and related security technologies should be applied throughout the data lifecycle (33), supported by strong encryption algorithms (101), data masking, and pseudonymization (45). Physical education teachers should participate in professional training to strengthen their algorithmic literacy and ethical awareness (35, 102, 103), incorporate privacy protection into professional ethics (104), educate students about ethical conduct in digital physical education (29), and improve their awareness of digital security risks (105). Students and parents should strengthen their capacity for data self-determination and provide stage-specific authorization through dynamic consent mechanisms. Together, these measures can establish a coordinated, multistakeholder system of data protection.

Second, adhere to algorithmic ethics principles, promote AI for good and transparent AI-enabled practices. AIPE should adhere to four fundamental principles: transparency, explainability, fairness, and safety. A top-level legislative framework grounded in human-centered ethics and algorithmic justice should be developed to ensure algorithmic robustness and sustainability (55). Education, sports, and cyberspace authorities could jointly establish a Youth Health Technology Ethics Committee, introduce algorithm registration and access requirements (35), and develop classification and functional evaluation standards for intelligent equipment used in school physical education, with algorithmic transparency specified as a core criterion (21). Regulatory authorities should oversee the entire process through which physical education teachers use AI and require appropriate measures to identify and alleviate bias in model-training data (106). An algorithmic power audit mechanism incorporating fairness and explainability should also be established (35). Civil society organizations can play a complementary role in this process. For example, the US-based nonprofit Algorithmic Justice League engages the public in addressing algorithmic discrimination and advocates the timely disclosure of technical and algorithmic standards (33). Schools should convene interdisciplinary experts to conduct regular algorithmic reviews, optimize models, and ensure the routine inspection and maintenance of AI platforms, thereby promoting algorithmic fairness (30). Anomalous data should automatically trigger human review (28). Clear accountability mechanisms should delineate the responsibilities of all relevant actors and be accompanied by risk-warning, appeal, and redress procedures. These measures would provide a transparent basis for tracing responsibility when AI-supported decisions in physical education are biased and ensure timely remedies when rights or interests are harmed (97). Physical education teaching and research groups should also establish reporting channels through which concerns about algorithmic bias or other ethical issues can be raised (87). Technology developers should strengthen their ethical competence and avoid embedding socially or educationally discriminatory assumptions in algorithm design (22). Explainable AIPE should be advanced through techniques such as Local Interpretable Model-agnostic Explanations (LIME) and SHapley Additive exPlanations (SHAP), supporting the development of physical education systems that respond to students’ needs while meeting privacy requirements (22, 107). To address AI hallucinations, human-in-the-loop mechanisms should incorporate physical education teachers’ contextual judgments into algorithmic calibration and require human verification of AI-generated content, thereby preserving human agency in knowledge construction (28). Physical education teachers should draw on their distinctive strengths in value guidance and emotional engagement (55) and integrate AI ethics into physical education curricula (108). Students should cross-check AI outputs using multiple tools (68) rather than accepting algorithmic recommendations uncritically (55). Parents should encourage children to move beyond data-oriented evaluation, recognize the intrinsic value of physical education (74), and develop critical thinking skills (20).

#### 4.2.2 Strategies for the physical education dimension

First, preserve diversity in physical education teaching and student development to prevent their homogenization. The epistemic constraints imposed by dataism should be critically examined, and the data literacy of stakeholders involved in school physical education governance should be strengthened (62). Human–AI collaboration should be used to align precision in teaching with the holistic development of students (65). Governments should allocate dedicated resources to support the development of locally grounded physical education curricula that reflect national and regional cultural traditions, while incorporating creative bodily expression into evaluation standards (60). Schools should establish tiered AI literacy standards (109) and provide regular training centered on ethical responsibility to improve physical education teachers’ intelligent digital literacy (29, 80, 83, 110). Accelerating teachers’ intelligent digital transformation would also strengthen the resilience of AI-enabled physical education to emerging risks (25). Talent development models should be reconsidered to address the tensions introduced by intelligent digital physical education (111). Algorithm design should accommodate creative movement rather than privilege uniform performance standards (74). Drawing on learning analytics, technology developers could build large models capable of dynamically adapting physical education content, enabling personalized recommendations and the adaptive matching of task difficulty (112). Physical education teachers should implement differentiated teaching and individualized guidance according to students’ physical conditions and interests (76). Students, in turn, should respect their own movement preferences and distinctive forms of bodily expression rather than conform uncritically to algorithmic standards (55).

Second, maintain meaningful educational relationships and the professional agency of physical education teachers, while promoting authentic teacher-student and student–student interactions and humanistic care. Addressing alienation in teacher-student and student–student relationships requires reaffirming the central role of physical education teachers in value guidance and emotional interaction (58) and adopting a human teacher-led, technology-assisted model of human–AI collaboration (49, 100). Technology developers should integrate multimodal emotion-recognition technologies to help teachers identify affective signals during physical activity, thereby supporting rather than replacing teachers’ emotional communication with students (21). Physical education teachers should actively increase the frequency and depth of their interactions with students (55), incorporate affective development into course assessment, and deliberately establish technology-free periods during lessons. By disengaging from digital devices and participating in face-to-face interaction through bodily co-presence, students can be guided to reflect, engage emotionally, and rebuild trust with teachers and peers (28). The relationship between intelligent technologies and physical education teaching must also be clearly defined: physical education teaching should remain primary, with intelligent technologies serving an auxiliary role. This distinction is necessary to prevent technological overreach and the resulting alienation in teacher-student relationships (113). Students should also recognize the limitations of AI in providing emotional support and actively participate in authentic social interactions (56).

Third, uphold the goals and humanistic values of physical education to prevent technological logic from displacing educational logic and value judgment. Addressing deviation from the goals of physical education, lack of humanistic care, and value alienation requires adherence to the value-rational principle that humans must remain in control of machines, with reference to the Ethics of Artificial Intelligence in Education: A Reference Framework (114). Governments should issue ethical guidelines for AIPE and establish market-entry and tiered approval systems (22). On this basis, schools should establish ethics review committees, implement an ideological and political education review chain and a circuit-breaker mechanism, and prohibit AI from fully replacing teachers in classroom value guidance or automatically assessing students’ moral and ideological development (38). Technology developers should disclose the sources and limitations of training data to improve system transparency (22). Physical education teachers should retain AI-free components in teaching and preserve final decision-making authority to ensure that AI serves the educational mission of physical education (56). They should also strengthen their AI literacy to identify and alleviate potential bias (115). Students and parents should move beyond evaluation based solely on test scores and quantitative performance data, and recognize the broader value of physical education in character formation and the development of cooperative values (65, 76).

Fourth, strengthen academic integrity governance and regulate the use of AI in learning, teaching, and research. Clear academic standards are essential to prevent the distortion of knowledge production. Schools should define the permissible and prohibited uses of AI in academic activities, establish human–AI collaboration ethics committees, prohibit the direct adoption of AI-generated research hypotheses or the wholesale delegation of academic tasks to algorithms (28), and introduce disclosure requirements for research outputs together with blockchain-based provenance systems (68). Physical education teachers should revise assessment practices by increasing the use of process-based evaluation, such as practice logs and on-site skill assessments (43). Students should comply with academic standards, clearly disclose the stages at which AI was used, and avoid plagiarism and data fabrication, thereby safeguarding academic rigor and ensuring accountability and traceability (28).

#### 4.2.3 Strategies for the body dimension

First, clarify body boundaries and anchor identity formation. Clear limits on human-machine symbiosis are needed to prevent digital embodiment from weakening human agency, embodied experience, and student identity (116). Governments should define ethical limits for wearable devices, virtual reality, and brain-computer interfaces, while mandating bodily data anonymization and device approval standards (48, 75). Schools should establish psychological support systems, identify excessive dependence on technologically mediated embodiment, and use real-world physical activities to restore bodily awareness (65). Developers should incorporate “reality-anchoring” functions after virtual exercise. Physical education teachers should strengthen identity and boundary education, clarify the differences between virtual and real environments, and organize technology-free activities grounded in bodily co-presence (55, 88). Students should regard data as tools rather than the essence of the body and prevent virtual personas from overshadowing their authentic selves (48). Parents should encourage outdoor activity in natural environments to reinforce physical-world cognition and authentic self-awareness (35, 74).

Second, safeguard body meaning by preserving authentic bodily practice. Governments should set minimum requirements for direct and nature-based physical activity, regulate adolescents’ use of intelligent devices, and ensure that algorithmic decisions protect bodily autonomy, dignity, and holistic development (31, 35). Schools should monitor screen time and internet addiction risks through AI-assisted databases while limiting virtual reality to supplementary use so that it expands rather than replaces embodied experience (26, 86). Developers should prioritize perception-enhancing technologies and adopt a humanistic, inclusive approach to body-related innovation (77, 88). Physical education teachers should include technology-free periods, face-to-face interaction, and reflection on movement experiences while accommodating diverse bodily needs (28, 48). Students and parents should prioritize authentic movement settings and prevent virtual practice from displacing real-world bodily experience.

Third, reassess body value and restore the educational essence of physical education. Technology should serve, rather than define or displace, bodily value, allowing physical education and technology to coexist without subordination (65). Governments should regulate excessive technological intervention and use embodied, living, and ethical development to define their legitimate boundaries (48). Schools should include non-quantifiable indicators, such as sports ethics and teamwork, in assessment and preserve teachers’ final discretion in ethical and safety decisions (28, 40). Technology developers should design systems based on health rather than efficiency; avoid promoting extreme data competition or exam-cheating technologies. Physical education teachers should cultivate character and willpower, reduce dependence on data-based performance indicators, and restore students from “data objects” to “developing subjects” (35, 76, 79, 83). Students should view sports as a means of growth and enjoyment, rather than as a means of achieving data targets, and should value achievement and development throughout the entire physical education process. Parents should move beyond score-centered evaluation and encourage authentic physical activity, thereby recovering its broader educational value (77).

### 4.3 Public health implications of ethical risks in AIPE

The ethical risks identified in this review have public health relevance because physical education is an important school-based setting for promoting physical activity, psychosocial wellbeing, and lifelong health-related behaviors (117, 118). However, our findings do not establish a direct causal relationship between AI-related ethical risks and population-level health outcomes. Rather, they suggest plausible pathways through which these risks may affect equitable, safe, and health-promoting participation in physical education.

First, algorithmic bias, algorithmic limitations, and homogenized teaching may undermine equitable participation. AI systems based on unrepresentative data or standardized performance models may provide less appropriate support to students with different physical abilities, socioeconomic backgrounds, or learning needs, potentially reinforcing inequalities in school-based health promotion (7, 119, 120). Second, privacy infringement, data leakage, and intensive digital monitoring may affect psychosocial wellbeing and bodily autonomy. Continuous collection and algorithmic interpretation of biometric, physiological, and behavioral data may increase perceptions of surveillance, psychological pressure, concerns about fairness and exclusion, and negative effects on self-perception (7, 121). The body-related risks identified in this review are also consistent with broader concerns regarding psychological privacy, bodily datafication, and autonomy, although such evidence does not directly validate these themes (121, 122). These concerns highlight the importance of informed consent, data minimization, safety safeguards, and meaningful student autonomy. Third, differences in technological access, digital literacy, data protection capacity, and algorithmic representation may create unequal benefits and disproportionate exposure to harm across schools and student groups, thereby contributing to digital and data-related health inequities (7, 120, 123). Finally, privacy breaches, opaque algorithms, inaccurate assessments, and excessive replacement of teacher judgment may weaken trust in AI-enabled educational and health-promoting technologies. Responsible implementation therefore requires transparent data governance, algorithmic accountability, human oversight, and stakeholder participation (120, 124).

Overall, the public health significance of these findings lies primarily in safeguarding equitable, safe, autonomous, and health-promoting participation in physical education rather than claiming direct downstream population-health effects. Future research should examine how these risks influence physical activity, psychosocial wellbeing, autonomy, trust, and health equity over time.

## 5 Conclusion and limitations

Using a mixed deductive-inductive thematic analysis, this study developed a three-dimensional framework of AIPE ethical risks across the technology, physical education, and body dimensions. The framework reveals a cascading process in which technological risks extend into physical education and ultimately affect the body, forming systemic risks through interactions among the three dimensions. The study also proposes stakeholder-coordinated alleviation strategies centered on trustworthy AI, integrating technological governance, ethical restoration in physical education, and protection of bodily subjectivity. By incorporating the body dimension, it extends the theoretical scope of AIPE ethics and provides a systematic foundation for trustworthy, human-centered, and sustainable development. From a public health perspective, these findings highlight the importance of ethical AI governance in safeguarding equitable, safe, autonomous, and health-promoting participation in physical education, with potential implications for physical activity participation, psychosocial wellbeing, bodily autonomy, and digital health equity. Rather than implying direct population-level health effects, the framework identifies pathways through which responsible AIPE may contribute to more inclusive and health-supportive educational environments.

Despite its contributions, this systematic review has several limitations. First, although six major academic databases were searched and supplemented by reference searching, relevant studies indexed elsewhere or published in non-indexed sources may have been missed. Second, only peer-reviewed journal articles in both Chinese and English were included, while conference proceedings, dissertations, preprints, and other gray literature were excluded. These restrictions may have introduced language and publication bias and limited the comprehensiveness of the evidence base. Third, among the 92 studies included in this review, 74 were qualitative. Although the predominance of qualitative research provided rich contextual insights into ethical risks associated with AIPE, the limited availability of controlled intervention studies and large-sample empirical research constrained the quantitative validation of the findings and the ability to draw causal inferences. Fourth, the geographic distribution was highly concentrated, with most studies conducted in China, which may limit the transferability of the findings across different educational, technological, cultural, and regulatory contexts. Finally, many mitigation strategies identified in the literature were proposed or recommended rather than empirically evaluated. Their feasibility, effectiveness, and potential unintended consequences therefore require further investigation through quantitative, longitudinal, mixed-methods, and intervention-based research.

## 6 Future research directions

Future research should advance in three directions. First, ethical assessment and standard development should be strengthened by creating comprehensive, standardized tools that evaluate both the likelihood of AIPE risks and their potential harm to stakeholders. An indicator system and application guidelines should also be established to ensure institutional ethical compliance. Second, empirical validation should be reinforced. The proposed alleviation strategies require further refinement and empirical validation across different schools, regions, and educational contexts. Systematic surveys should examine stakeholder needs and implementation barriers, while meta-analyses and longitudinal studies should clarify the long-term effects of ethical risks and alleviation measures. Third, international cooperation and prospective inquiry should be expanded. A global AIPE ethics platform could compile ethical cases and best practices to support knowledge sharing and collaborative governance. Emerging risks associated with generative AI, embodied intelligence, and related technologies should also be integrated into ongoing research and international agendas, supporting a responsible, trustworthy, and ethically sound AIPE ecosystem.

## Data Availability

The original contributions presented in the study are included in the article/Supplementary material, further inquiries can be directed to the corresponding author.
