# Supplementary material for: Identifying and alleviating ethical risks of artificial intelligence in physical education: a systematic review
Source: Front Public Health. 2026 Sep 10;14:1945681. doi: 10.3389/fpubh.2026.1945681 (PMC13601240; doi:10.3389/fpubh.2026.1945681)
Supplement: Supplementary file 1 [file Data_Sheet_1.docx]

Supplementary Materials

# Supplementary material 1 The search strategies for each database

**1. SpringerLink search algorithm: 81**

Retrieval date: June 15, 2026

(Used the following Filters: Content type: Article; Languages: English; Publishing model: Open access)

((“physical education” OR “physical education teacher*” OR “physical education teaching” OR “physical education class” OR “physical education curriculum” OR “school physical education” OR “sport education” OR “sport pedagogy”) AND (“Artificial Intelligence” OR AI OR “Machine Learning” OR “Deep Learning” OR ChatGPT OR “Generative AI” OR “Artificial Neural Network” OR “Convolutional Neural Network” OR “Recurrent Neural Network” OR “Decision Tree” OR “Naive Bayes model” OR “Natural Language Processing” OR “image processing” OR “Logic programming” OR “fuzzy logic” OR “Computational Vision” OR “face recognition” OR “Voice recognition” OR “Knowledge Graph” OR “Multimodal Models” OR “Situation Awareness” OR “learning analytics” OR “data mining” OR “adaptive learning” OR “personalized learning” OR “intelligent tutoring system” OR “student model” OR “learner model” OR “prediction model” OR “prediction system” OR “feedback system” OR “recommended system” OR “virtual agent” OR “conversational agent” OR chatbot OR “Expert system”) AND (ethic* OR “ethical risk*” OR “ethical issue*” OR “ethical challenge*” OR “ethical concern*” OR “ethical implication*” OR “algorithmic bias” OR fairness OR privacy OR “data security” OR surveillance OR accountability OR responsibility OR transparency OR explainability OR trustworthiness OR trustworthy OR governance OR regulation OR “risk mitigation”))

**2. Web of Science search algorithm: 74**

Retrieval date: June 15, 2026

(Refined by the following: Languages: English)

TS=((“physical education” OR “physical education teacher*” OR “physical education teaching” OR “physical education class” OR “physical education curriculum” OR “school physical education” OR “sport education” OR “sport pedagogy”) AND (“Artificial Intelligence” OR AI OR “Machine Learning” OR “Deep Learning” OR ChatGPT OR “Generative AI” OR “Artificial Neural Network” OR “Convolutional Neural Network” OR “Recurrent Neural Network” OR “Decision Tree” OR “Naive Bayes model” OR “Natural Language Processing” OR “image processing” OR “Logic programming” OR “fuzzy logic” OR “Computational Vision” OR “face recognition” OR “Voice recognition” OR “Knowledge Graph” OR “Multimodal Models” OR “Situation Awareness” OR “learning analytics” OR “data mining” OR “adaptive learning” OR “personalized learning” OR “intelligent tutoring system” OR “student model” OR “learner model” OR “prediction model” OR “prediction system” OR “feedback system” OR “recommended system” OR “virtual agent” OR “conversational agent” OR chatbot OR “Expert system”) AND (ethic* OR “ethical risk*” OR “ethical issue*” OR “ethical challenge*” OR “ethical concern*” OR “ethical implication*” OR “algorithmic bias” OR fairness OR privacy OR “data security” OR surveillance OR accountability OR responsibility OR transparency OR explainability OR trustworthiness OR trustworthy OR governance OR regulation OR “risk mitigation”))

**3. EBSCOhost (SPORTDiscus with Full Text, Academic Search Premier) search algorithm: 13**

Retrieval date: June 15, 2026

(Refined by the following: Scource Types: Academic Journals; Languages: English; full text; peer review)

TI ( ((“physical education” OR “physical education teacher*” OR “physical education teaching” OR “physical education class” OR “physical education curriculum” OR “school physical education” OR “sport education” OR “sport pedagogy”) AND (“Artificial Intelligence” OR AI OR “Machine Learning” OR “Deep Learning” OR ChatGPT OR “Generative AI” OR “Artificial Neural Network” OR “Convolutional Neural Network” OR “Recurrent Neural Network” OR “Decision Tree” OR “Naive Bayes model” OR “Natural Language Processing” OR “image processing” OR “Logic programming” OR “fuzzy logic” OR “Computational Vision” OR “face recognition” OR “Voice recognition” OR “Knowledge Graph” OR “Multimodal Models” OR “Situation Awareness” OR “learning analytics” OR “data mining” OR “adaptive learning” OR “personalized learning” OR “intelligent tutoring system” OR “student model” OR “learner model” OR “prediction model” OR “prediction system” OR “feedback system” OR “recommended system” OR “virtual agent” OR “conversational agent” OR chatbot OR “Expert system”) AND (ethic* OR “ethical risk*” OR “ethical issue*” OR “ethical challenge*” OR “ethical concern*” OR “ethical implication*” OR “algorithmic bias” OR fairness OR privacy OR “data security” OR surveillance OR accountability OR responsibility OR transparency OR explainability OR trustworthiness OR trustworthy OR governance OR regulation OR “risk mitigation”)) OR AB ((“physical education” OR “physical education teacher*” OR “physical education teaching” OR “physical education class” OR “physical education curriculum” OR “school physical education” OR “sport education” OR “sport pedagogy”) AND (“Artificial Intelligence” OR AI OR “Machine Learning” OR “Deep Learning” OR ChatGPT OR “Generative AI” OR “Artificial Neural Network” OR “Convolutional Neural Network” OR “Recurrent Neural Network” OR “Decision Tree” OR “Naive Bayes model” OR “Natural Language Processing” OR “image processing” OR “Logic programming” OR “fuzzy logic” OR “Computational Vision” OR “face recognition” OR “Voice recognition” OR “Knowledge Graph” OR “Multimodal Models” OR “Situation Awareness” OR “learning analytics” OR “data mining” OR “adaptive learning” OR “personalized learning” OR “intelligent tutoring system” OR “student model” OR “learner model” OR “prediction model” OR “prediction system” OR “feedback system” OR “recommended system” OR “virtual agent” OR “conversational agent” OR chatbot OR “Expert system”) AND (ethic* OR “ethical risk*” OR “ethical issue*” OR “ethical challenge*” OR “ethical concern*” OR “ethical implication*” OR “algorithmic bias” OR fairness OR privacy OR “data security” OR surveillance OR accountability OR responsibility OR transparency OR explainability OR trustworthiness OR trustworthy OR governance OR regulation OR “risk mitigation”)) )

**4. ScienceDirect search algorithm: 348**

Retrieval date: June 15, 2026

(Used the following Filters: Article type: Research articles; Access type: open access and open archive)

((“physical education” OR “physical education teaching”) AND (“Artificial Intelligence” OR AI OR “Generative AI”) AND (ethic OR “ethical risk” OR “ethical issue”))

**5. Scopus search algorithm:** **166**

Retrieval date: June 15, 2026

(Refined by the following: Languages: English)

TITLE-ABS-KEY=((“physical education” OR “physical education teacher*” OR “physical education teaching” OR “physical education class” OR “physical education curriculum” OR “school physical education” OR “sport education” OR “sport pedagogy”) AND (“Artificial Intelligence” OR AI OR “Machine Learning” OR “Deep Learning” OR ChatGPT OR “Generative AI” OR “Artificial Neural Network” OR “Convolutional Neural Network” OR “Recurrent Neural Network” OR “Decision Tree” OR “Naive Bayes model” OR “Natural Language Processing” OR “image processing” OR “Logic programming” OR “fuzzy logic” OR “Computational Vision” OR “face recognition” OR “Voice recognition” OR “Knowledge Graph” OR “Multimodal Models” OR “Situation Awareness” OR “learning analytics” OR “data mining” OR “adaptive learning” OR “personalized learning” OR “intelligent tutoring system” OR “student model” OR “learner model” OR “prediction model” OR “prediction system” OR “feedback system” OR “recommended system” OR “virtual agent” OR “conversational agent” OR chatbot OR “Expert system”) AND (ethic* OR “ethical risk*” OR “ethical issue*” OR “ethical challenge*” OR “ethical concern*” OR “ethical implication*” OR “algorithmic bias” OR fairness OR privacy OR “data security” OR surveillance OR accountability OR responsibility OR transparency OR explainability OR trustworthiness OR trustworthy OR governance OR regulation OR “risk mitigation”))

**6. CNKI search algorithm: 1120**

Retrieval date: June 15, 2026

(Used the following Filter: Source category: PKU Core and CSSCI)

SU = (“physical education” OR “physical education teaching” OR “school physical education” OR “physical education teacher” OR sport OR “smart sports” OR “intelligent sports”) AND SU = (“artificial intelligence” OR AI OR “generative artificial intelligence” OR GAI OR GenAI OR “deep learning” OR “machine learning” OR ChatGPT OR DeepSeek OR “digital-intelligence technology” OR “digital-intelligent transformation” OR “large model” OR Sora OR wearable OR “digital technology” OR digitalization OR “intelligent technology” OR “intelligent era”)-(In Chinese)

# Supplementary material 2 The list for the included studies

1. Liu B, Wang WL. Limitations, theoretical origins, and resolution strategies of multidimensional reflection on the popularity of artificial intelligence in physical education. J Shanghai Univ Sport. (2025) 49:13-23. doi: 10.16099/j.sus.2025.07.14.0005
2. Lee HS, Lee J. Applying artificial intelligence in physical education and future perspectives. Sustainability. (2021) 13:351. doi: 10.3390/su13010351
3. Chen Q, Ping B. Research on physical education teaching management based on the “Internet+” platform. Int J Healthc Inf Syst Inform. (2025) 20:1-20. doi: 10.4018/IJHISI.383510
4. Guo JH, Xiong W. Artificial intelligence in the field of physical education: connotation, ethical risks, and mitigation strategies. J Sports Res. (2025) 39:21-31. doi: 10.15877/j.cnki.nsic.20250123.002
5. Jia Z, Tang D, Mei L, Lu X, Bai X. Reshaping teachers’ roles in intelligent physical education and job crafting: a multimethod approach. J Vis Exp. (2026) 227:e69591. doi: 10.3791/69591
6. Ye SD, Duan R. Key dimensions, constraints and practical approaches of AI empowering equity in physical education. J Shenyang Sport Univ (2024) 43:43-50. doi: 10.12163/j.ssu.20240688
7. Bofill-Herrero A, García-Taibo O, Ferriz-Valero A, Baena-Morales S. Exploring the role and challenges of AI tools in physical education: qualitative insights from in-service, trainee, and future teachers. Sport Educ Soc. (2025):1-16. doi: 10.1080/13573322.2025.2547869
8. Hu T, Min H. Application of generative artificial intelligence empowering professional independent development of physical education teachers. J Shenyang Sport Univ (2025) 44:59-65. doi: 10.12163/j.ssu.20241159
9. Yin HG, Tang MG. The teaching application and ethical review of DeepSeek knowledge distillation technology in the construction of ideological and political education in college physical education courses. J Sports Res (2026) 40:87-96. doi: 10.15877/j.cnki.nsic.20250904.001
10. Zhang XL, Yao SQ, Zhou Y, Huo AB, Zhang L, Mao ZM. Application scenarios and key technological issues of artificial intelligence promoting the digital transformation of school sports. J Sports Res (2025) 39:11-20. doi: 10.15877/j.cnki.nsic.20250307.001
11. Wang GL, Song DT, Wang F, Zhang NX. Practical dilemmas and relief pathways of intelligent physical education in China. J Xi’an Phys Educ Univ. (2024) 41:544-552. doi: 10.16063/j.cnki.issn1001-747x.2024.04.013
12. Guo JH. Technology, ethics and law: a triple survey of the application of virtual digital humans in the field of sports. J Shenyang Sport Univ (2024) 43:131-137. doi: 10.12163/j.ssu.20240666
13. Cao BC, Xu RH, Zhou XM, Li LT. Key driving pathways for collaborative development of youth and artificial intelligence in Chinese sports modernization. J Capital Univ Phys Educ Sports. (2025) 37:618-627. doi: 10.14036/j.cnki.cn11-4513.2025.06.004
14. Duan L. Obstacles and driving forces of digital transformation of school physical education under education digitalization strategy. J Shenyang Sport Univ (2025) 44:37-43. doi: 10.12163/j.ssu.20241464
15. Zhou H, Jiang J, Li HY, Wu B. Value, risks, and pathways of generative artificial intelligence empowering the development of physical education. Sports Cult Guide. (2025):96-103.
16. Wang JH, Zhao FX. Technical concerns and prevention strategies of integrating generative artificial intelligence in physical education. J Shenyang Sport Univ. (2025) 44:30-37. doi: 10.12163/j.ssu.20250581
17. Li SJ, Li W, Yang T. Bridging the digital divide: construction and enhancement of digital literacy among primary and secondary school physical education teachers. Educ Acad Mon. (2024):85-93. doi: 10.16477/j.cnki.issn1674-2311.2024.12.004
18. Dong Q, Liu Y. Digital transformation of education empowering the development of school physical education: history, current status, and future prospects. China Sport Sci. (2024) 44:50-60. doi: 10.16469/J.css.2024KX018
19. Martín-Rodríguez A, Madrigal-Cerezo R. Technology-enhanced pedagogy in physical education: bridging engagement, learning, and lifelong activity. Educ Sci. (2025) 15:409. doi: 10.3390/educsci15040409
20. Chen Y, Xian D, Zhao Y, Sun Y, Ren Y, Wang C. AI-enabled learning analytics use relates to physical literacy and engagement in university PE via smart teaching and personalised feedback. Sci Rep. (2026) 16:8341. doi: 10.1038/s41598-026-39778-9
21. Zhang XM, Zhu Q, Cai YJ, Lu GF, Wang JY. Application scenarios, risks, and solutions of artificial intelligence empowering physical education assessment. J Sports Res. (2024) 38:38-49. doi: 10.15877/j.cnki.nsic.20240527.002
22. Yuan SW, Mao J, Fu ZH, Ge DD. Transformation of physical education evaluation empowered by digital technology: connotation, motivations, problems, and strategies. J Shenyang Sport Univ. (2024) 43:31-38. doi: 10.12163/j.ssu.20240152
23. Cui JN, Wang L, Liu JX, Feng XL, Zhang KB. Ethical concerns and trustworthy governance pathways for applying artificial intelligence to promote adolescent physical health. China Sport Sci. (2026) 46:51-62. doi: 10.16469/J.css.2026KX005
24. Chang SH, Chen SY, Chang CH. Exploring opportunities and challenges toward ChatGPT for inclusion in sport education. J Hosp Leis Sport Tour Educ (2025) 37:100572. doi: 10.1016/j.jhlste.2025.100572
25. Konukman F, Sortwell A, Filiz B, Tüfekçioglu E, Yılmaz EB, Ünlü H. Using artificial intelligence in teaching health and physical education. J Phys Educ Recreat Dance. (2025) 96:58-62. doi: 10.1080/07303084.2025.2522601
26. Genç N. Artificial intelligence in physical education and sports: new horizons with ChatGPT. Mediterr J Sport Sci. (2023) 6:17-32. doi: 10.38021/asbid.1291604
27. Albaloul O, Marttinen R, Killian C. Unlocking educational potential: how physical education teachers can thoughtfully benefit from using ChatGPT for planning, instruction, and assessment. J Phys Educ Recreat Dance. (2024) 95:32-41. doi: 10.1080/07303084.2024.2378776
28. Guo JH, Xiong W. Research limits and beyond of artificial intelligence empowering school physical education: an investigation based on technical phenomenology. J Phys Educ (2025) 32:87-96. doi: 10.16237/j.cnki.cn44-1404/g8.20250324.008
29. Wu S, Jin HX. Digital and intelligent governance of high-quality and balanced development of school physical education in China. J Shenyang Sport Univ (2024) 43:29-35. doi: 10.12163/j.ssu.20240734
30. Dergaa I, Chamari K, Zmijewski P, Ben Saad H. From human writing to artificial intelligence-generated text: examining the prospects and potential threats of ChatGPT in academic writing. Biol Sport. (2023) 40:615-622. doi: 10.5114/biolsport.2023.125623
31. Killian CM, Marttinen R, Howley D, Sargent J, Jones EM. “Knock, knock... who’s there?” ChatGPT and artificial intelligence-powered large language models: reflections on potential impacts within health and physical education teacher education. J Teach Phys Educ. (2023) 42:385-389. doi: 10.1123/jtpe.2023-0058
32. Jiang JJ, Li CX, Yin ZH, Liu HH. Diverse models and evolving paradigms: current development and future prospects of large sports models. J Chengdu Sport Univ. (2026) 52:50-62. doi: 10.15942/j.jcsu.2026.01.06
33. Li XT, Hu LP, Shang XM, Chen RZ. Reconstructing the paradigm of sports science research through large language models: applications and insights—A case study of ChatGPT. J Xi’an Phys Educ Univ. (2023) 40:405-415. doi: 10.16063/j.cnki.issn1001-747x.2023.04.003
34. Cheng WG, Wang JH. Digital transformation of school physical education in China: prospects, dilemmas, and pathways. J Xi’an Phys Educ Univ. (2025) 42:613-622. doi: 10.16063/j.cnki.issn1001-747x.2025.05.011
35. Zhou SW, Cheng CY. Body and technology: ethical consideration of artificial intelligence in sports from the perspective of body philosophy. J Shanghai Univ Sport. (2021) 45:1-11. doi: 10.16099/j.sus.2021.09.001
36. Du XJ, Qin HJ. Integration of artificial intelligence and physical education teaching from the perspective of embeddedness theory: forms, dilemmas, and pathways. J Beijing Sport Univ. (2025) 48:131-142. doi: 10.19582/j.cnki.11-3785/g8.2025.09.011
37. Baena-Morales S, Sánchez-Jarque M, Ferriz-Valero A, Bofill-Herrero A. The paradox of experience: generational analysis of AI integration in physical education using the iTPACK framework. Phys Educ Sport Pedagogy. (2025):1-22. doi: 10.1080/17408989.2025.2536145
38. Wang J. Implementation framework and development ideas for smart physical education teaching. Sports Cult Guide. (2023):103-110.
39. Yang JZM, Wang ZH. Seeking change amid change: reflections on sports science in the ChatGPT era—A qualitative study based on the workshop “Academic research on sports in the ChatGPT era: body as method”. Sports Sci. (2023) 44:1-8. doi: 10.13598/j.issn1004-4590.2023.02.001
40. Yang Y, Ren J. Seventy years of physical education curriculum reform in primary and secondary schools in China: concurrently discussing the influence of artificial intelligence on physical education. China Sport Sci. (2020) 40:32-37. doi: 10.16469/j.css.202006005
41. Liao YS, Feng XL, Wang XL. The shackles of the “digital Leviathan” in the digital transformation of school physical education and its dissolution. J Phys Educ. (2025) 32:10-20. doi: 10.16237/j.cnki.cn44-1404/g8.2025.06.005
42. Zhang XM, Zhu Q, Cai YJ. Transformation of physical education learning in the era of artificial intelligence: typical patterns, practical challenges, and breakthrough pathways. J Shanghai Univ Sport. (2025) 49:37-48. doi: 10.16099/j.sus.2025.02.23.0001
43. Liu W, Peng J, Zhou BY. Value analysis, realistic examination and approach analysis of artificial intelligence into physical education. J Shenyang Sport Univ. (2023) 42:61-67. doi: 10.12163/j.ssu.20230637
44. Gamlem SM, Johnston S, Moltudal S, McGrane J, Hopfenbeck TN. Generative AI in initial teacher education: exploring the alignment of perceptions and experiences of pre-service teachers and their teacher educators. Teach Teach Educ. (2026) 172:105382. doi: 10.1016/j.tate.2026.105382
45. Feng T, Zhao FX. Mechanisms and empowerment limits of generative artificial intelligence driving the transformation of teaching and research paradigms for college physical education teachers under human-machine collaboration. J Sports Res. (2026) 40:92-103. doi: 10.15877/j.cnki.nsic.20251028.001
46. Wang NN. Theoretical and practical study on artificial intelligence empowering professional development of physical education teachers in primary and secondary schools. J Shenyang Sport Univ. (2025) 44:23-29. doi: 10.12163/j.ssu.20250744
47. Wang JH, Dong H. High-quality development pathways for youth sport and health empowered by artificial intelligence. J Beijing Sport Univ. (2024) 47:1-11. doi: 10.19582/j.cnki.11-3785/g8.2024.04.001
48. Guo JH, Xu YM, Xue ZM, Gao S. Toward education digitization: metaverse physical teaching’s functional scenarios and development direction. Sports Sci. (2025) 46:62-71. doi: 10.13598/j.issn1004-4590.2025.01.007
49. Lv K, Tang Y. Empowering high-quality development of youth sports through digital technology: challenges and solutions. J Beijing Sport Univ. (2024) 47:49-59. doi: 10.19582/j.cnki.11-3785/g8.2024.04.005
50. Qiu FH, Zhang SW. Exploring pathways of empowering reform of physical education evaluation through digital intelligence technology under the student physical fitness enhancement program. J Shenyang Sport Univ (2026) 45:44-50. doi: 10.12163/j.ssu.20260121
51. Ha T, Yu H, Lim B, Jung HY, Brooks C, Krause J, et al. Using ChatGPT in the field of kinesiology: opportunities and considerations. J Phys Educ Sport. (2024) 24:3-12. doi: 10.7752/jpes.2024.01001
52. Yu SM, Wang XY. Re-understanding the essence of school physical education in the context of education digitalization. J Shanghai Univ Sport. (2025) 49:1-10. doi: 10.16099/j.sus.2024.12.31.0003
53. Tannoubi A, Geanta V, Ardelean V, Setiawan E, Azaiez F. Rethinking ethical governance of generative AI in sport pedagogy research: a discipline-sensitive perspective. Front Public Health. (2026) 14:1812516. doi: 10.3389/fpubh.2026.1812516
54. Xu ZX. Artificial intelligence-driven paradigm shifts in sports science: mechanisms, manifestations, and pathways. China Sport Sci. (2026) 46:31-42. doi: 10.16469/J.css.2026KX014
55. Wu C, Moorhouse BL, Wan Y, Wu M. Exploring PhD students’ utilization of generative AI in academic writing for publication purposes: insights for EAP. J Engl Acad Purp (2026) 79:101612. doi: 10.1016/j.jeap.2025.101612
56. Guo Q, Yang YX, Yuan WX. AI-empowered home-school co-education in primary and secondary school physical education under social space theory. J Shenyang Sport Univ (2026) 45:37-43. doi: 10.12163/j.ssu.20251562
57. Sun GB. Intelligent physical education teaching: functional value, risk reflection, and future directions. Sports Cult Guide. (2023):96-103
58. Tao YL, Wang YQ. Practical shifts, hidden dilemmas, and the quest for vitality in sports education in the intelligent era. China Sport Sci. (2025) 45:67-74. doi: 10.16469/J.css.2025KX099
59. Xiao Z, Guo W. Generative artificial intelligence in university sports training: a mixed-methods study. Int J Inf Educ Technol. (2026) 16:1153-1160. doi: 10.18178/ijiet.2026.16.5.2584
60. Yang P, Liu Y, Li Y. The mediating role of self-regulation in fostering intelligent-TPACK and ethics in physical education teacher education students. Sci Rep. (2025) 15:34126. doi: 10.1038/s41598-025-21795-9
61. Huang AF, Wang SC. Exploration on new-quality body characteristics of school physical education during the post-human era. J Phys Educ. (2024) 31:1-7. doi: 10.16237/j.cnki.cn44-1404/g8.2024.06.002
62. Li Y, Lang J, Chen JF, Wang CQ. Precise cultivation of core literacy in physical education enabled by AI. Teach Adm. (2025) 36:91-96.
63. Yan J. Generative AI-powered embodied cognitive turn in school physical education: real-world obstacles and solutions. J Guangzhou Sport Univ. (2025) 45:10-19. doi: 10.13830/j.cnki.cn44-1129/g8.2025.06.002
64. Østerlie O, Killian C. Becoming human with technology: rethinking digital practice in physical education. Learn Media Technol. (2026):1-13. doi: 10.1080/17439884.2026.2663160
65. Wang Y, Wang X. Artificial intelligence in physical education: comprehensive review and future teacher training strategies. Front Public Health. (2024) 12:1484848. doi: 10.3389/fpubh.2024.1484848
66. Can HC, Alas DK. The silent scream of digital future: exploring AI awareness and quiet quitting in Turkish teachers. Acta Psychol (2025) 260:105589. doi: 10.1016/j.actpsy.2025.105589
67. Yang Y. Connotation, dilemmas, and promotion strategies of physical education in the era of artificial intelligence. Sports Cult Guide. (2022):104-110
68. Jiang XC, Mo HQ, Tang CC. Content and thinking of AI technology empowering physical education classroom teaching evaluation. Teach Adm. (2024) 23:42-44. doi:10.26993/j.cnki.jxygl.2024.23.011
69. Zhao G, Xi Y. Breakthroughs, prospects, and hidden concerns: transformation and implications of AI technology intervention in school physical education. J Tianjin Univ Sport. (2023) 38:283-288. doi: 10.13297/j.cnki.issn1005-0000.2023.03.006
70. Vahdani M, Labbaf A, Cronin L. Optimizing the artificial intelligence revolution in physical education: strategies for integration, adoption, and risk mitigation. J Phys Educ Recreat Dance. (2026) 97:44-53. doi: 10.1080/07303084.2025.2611752
71. Cheng B, Wang ZJ. Dilemmas and survival of the inheritance of body techniques in the intelligence era. J Hunan Univ Sci Technol (Soc Sci Ed). (2020) 23:100-107. doi: 10.13582/j.cnki.1672-7835.2020.06.013
72. Bergdahl N, Sjöberg J. Attitudes, perceptions and AI self-efficacy in K-12 education. Comput Educ Artif Intell. (2025) 8:100358. doi: 10.1016/j.caeai.2024.100358
73. Yu HB, Wang ZH. With Sora coming, will the world of physical activities still be real? Research based on the workshop “Sora Tools and New Understanding of Sports Knowledge”. Sports Sci. (2024) 45:18-27. doi: 10.13598/j.issn1004-4590.2024.02.002
74. Li MC, Wang DE, Yuan Q, Qiu ZY, Zhou XC. Analysis of the reform of physical education teaching in colleges and universities empowered by digital technology. Sports Cult Guide. (2025):25-31.
75. Zamani A, Pourranjbar M. Faculty members’ lived experiences in using artificial intelligence to personalize sports programs: a phenomenological study. Res Sport Manag Mark. (2026) 7:83-94. doi: 10.22098/rsmm.2026.18648.1511
76. Wang JH, Cheng WG. Mechanisms and pathways of embedding digital-intelligence technology into the digital transformation and upgrading of school physical education. J Chengdu Sport Univ. (2025) 51:45-52. doi: 10.15942/j.jcsu.2025.04.06
77. Xu Y, Shao HT, Pu BW. Connotation, dilemmas, and pathways of digital empowerment for the high-quality development of university physical education. High Educ Explor. (2024) (4):117-121.
78. Jiang LL, Huang Q, Hou YY, Wang P. Functional mechanisms, application fields, and practical pathways of digital-intelligence technology empowering the modernization of school physical education. J Sports Res. (2023) 37:67-78. doi: 10.15877/j.cnki.nsic.20230828.001
79. Jiang JJ, Yin ZH, Zhou QY, Xu RL, Liao ZY, Sun MZ. Connotative characteristics, logical rationale, and practical approaches of artificial intelligence empowering physical education and health curriculum reform. J Shandong Sport Univ. (2025) 41:116-126. doi: 10.14104/j.cnki.1006-2076.2025.06.012
80. Jun W, Iqbal MS, Abbasi R, Omar M, Huiqin C. Web-semantic-driven machine learning and blockchain for transformative change in the future of physical education. Int J Semant Web Inf Syst (2024) 20:1-16. doi: 10.4018/IJSWIS.337961
81. Wang YS. Value implications and implementation pathways of digitalization empowering physical education teaching in universities. Theory Pract Educ. (2024) 44:57-60.
82. Li BC, Huang AF. Digital technology promoting the high-quality development of school physical education. Sports Cult Guide. (2025):97-103.
83. Jiang JJ, Yin ZH, Zhong QY, Guo Y, Sun MZ. Strategies of artificial intelligence empowering physical education and health interdisciplinary thematic learning. J Shenyang Sport Univ. (2025) 44:45-51. doi: 10.12163/j.ssu.20250447
84. Ma YH, Mu JW. Digitalization empowers professional development of physical education teachers: triple logic, implementation mechanisms, and pathway directions. J Guangzhou Sport Univ. (2025) 45:43-52. doi: 10.13830/j.cnki.cn44-1129/g8.2025.02.005
85. Li L, Li T, Liu B. Mechanisms, challenges, and breakthrough pathways of digital transformation in physical education classroom teaching: based on the perspective of TOE theory. J Shenyang Sport Univ. (2025) 44:23-29. doi: 10.12163/j.ssu.20241247
86. Li Y, Fayazi A, Zadeh HG. Federated knowledge graph-based hybrid personalized curriculum recommendation for privacy-preserving physical education teaching systems. J Cloud Comput. (2026) 15:1. doi: 10.1186/s13677-025-00812-2
87. Qin L, Liu Q, Yin ZH. Exploring pathways for reviewing and mitigating ethical risks of artificial intelligence in sport. J Chengdu Sport Univ. (2025) 51:34-41. doi: 10.15942/j.jcsu.2025.01.04
88. Liu B, Li CX, Wang S, Yu SY. Significance, mechanisms, and practical pathways of empowering the high-quality development of college sports with new quality productive forces. J Wuhan Sports Univ. (2024) 58:1-9. doi: 10.15930/j.cnki.wtxb.20240830.002
89. Zhang J. Reform and innovation of artificial intelligence technology for information service in university physical education. J Intell Fuzzy Syst. (2021) 40:3325-3335. doi: 10.3233/JIFS-189372
90. Liao QW. Digital textbooks for college physical education in the context of education digitalization: value implications, practical dilemmas, and implementation pathways. J Phys Educ. (2025) 32:74-80. doi: 10.16237/j.cnki.cn44-1404/g8.2025.05.016
91. Ouyang F, Du M. Evaluation of physical education teaching effectiveness empowered by intelligent technology: model construction and empirical testing. Shanghai Res Educ. (2023):41-47. doi: 10.16194/j.cnki.31-1059/g4.2023.07.011
92. Ompoc HMS, Aguinaldo JC. Evaluating the effectiveness of AI-driven applications in enhancing the physical fitness of tertiary physical education students. Int J Hum Mov Sports Sci. (2025) 13:74-82. doi: 10.13189/saj.2025.130109

# Supplementary material 3 Summary of screening decisions mapped to SPIDER elements across screening stages

| **Screening Stage** | **SPIDER Element Assessed** | **Records Retained (n)** | **Records Excluded (n)** | **Primary Exclusion Reasons (with SPIDER codes)** | **Stage Description** |
| --- | --- | --- | --- | --- | --- |
| Initial identification |  | 1,814 |  |  | Records identified through database searching (n = 1,802) and citation tracking (n = 12). |
| Duplicates & time-frame removal |  | 1,572 | 242 | Duplicates (76) / Outside predefined time frame (166) | Duplicates removed; records published outside the eligibility time window excluded. |
| Title screening | S (Sample) | 186 | 275 | S1 – Non-school contexts (208); S2 – Elite/professional sport or athletic training (35); S3 – Clinical/rehabilitation/healthcare settings (32) | Membership was fixed by the title-exclusion XML (n = 1,386); titles were then coded to one primary SPIDER reason. |
|  | PI (Phenomenon of Interest) |  | 1,087 | PI1 – No AI/AI-enabled technology (78); PI2 – No formal school PE/PE context (991); PI3 – Pure algorithm/model/system development without identifiable ethical relevance (18) | Title-only reasons were coded from the title evidence retained in the 1,572-record workbook. |
|  | D (Design) & E (Evaluation), preliminary |  | 24 | D/E-T – Effectiveness/performance orientation with no identifiable ethical or humanistic evaluation dimension (24) | D/E was used at title stage only when the technical/performance-only focus was self-evident. |
|  | Subtotal: Passed title screening | 186 | 1,386 |  | 1,572 − 1,386 = 186 records proceeded to abstract screening. |
| Abstract screening | S (Sample), re-check |  | 13 | S1 – Abstract confirmed a non-school/non-formal-PE context (13) | Membership was fixed by the abstract-exclusion XML (n = 72); population and setting were re-checked using abstract evidence. |
|  | PI (Phenomenon of Interest), re-check |  | 18 | PI1 – Abstract did not show substantive AI/AI-enabled technology (18) | AI and AIPE relevance were re-checked using abstract content. |
|  | D (Design), detailed preliminary assessment |  | 12 | D2 – Review/bibliometric/non-original evidence (1); D3 – Algorithm/model/system development lacking analyzable ethics-relevant original evidence (11) | Abstract evidence was used to identify ineligible designs or non-original studies. |
|  | E (Evaluation), detailed preliminary assessment |  | 29 | E1 – Effectiveness/performance/usability/efficiency outcomes only (1); E2 – General risk/challenge mention without an eligible ethics evaluation (20); E3 – No ethical risk, stakeholder impact, cause, or governance/mitigation analysis (8) | Ethics relevance required substantive assessment, not a passing or generic mention. |
|  | Subtotal: Passed abstract screening | 114 | 72 |  | 186 − 72 = 114 records proceeded to full-text review. |
| Full-text screening | D (Design), final verification | 114 | 0 |  | All 114 passed final D verification. |
|  | E (Evaluation), final verification | 114 | 0 |  | All 114 passed final E verification. |
|  | R (Research type) | 92 | 22 | R – Conference papers (16); systematic reviews (3); scoping review (1); editorials (2) | These 22 records passed title and abstract screening but were excluded at full text for the R criterion. |
| Final included |  | 92 |  |  | Studies meeting all SPIDER-based eligibility criteria and included in the final synthesis. |

# Supplementary material 4 Mixed Methods Appraisal Tool (MMAT), version 2018

| **Category of study designs** | **Methodological quality criteria** |
| --- | --- |
| Screening questions (for all types) | S1. Are there clear research questions? |
|  | S2. Do the collected data allow to address the research questions? |
| 1.Qualitative | 1.1. Is the qualitative approach appropriate to answer the research question? |
|  | 1.2. Are the qualitative data collection methods adequate to address the research questions? |
|  | 1.3. Are the findings adequately derived from the data? |
|  | 1.4. Is the interpretation of results sufficiently substantiated by data? |
|  | 1.5. Is there coherence between qualitative data sources, analysis and interpretation? |
| 2.Quantitative randomized controlled trials | 2.1. Is randomization appropriately performed? |
|  | 2.2. Are the groups comparable at baseline? |
|  | 2.3. Are there complete outcome data? |
|  | 2.4. Are outcome assessors blinded to the intervention provided? |
|  | 2.5 Did the participants adhere to the assigned intervention? |
| 3.Quantitative non-randomize | 3.1. Are the participants representative of the target population? |
|  | 3.2. Are measurements appropriate regarding both the outcome and intervention (or exposure)? |
|  | 3.3. Are there complete outcome data? |
|  | 3.4. Are the confounders accounted for in the design and analysis? |
|  | 3.5. During the study period, is the intervention administered (or exposure occurred) as intended? |
| 4.Quantitative descriptive | 4.1. Is the sampling strategy relevant to address the research question? |
|  | 4.2. Is the sample representative of the target population? |
|  | 4.3. Are the measurements appropriate? |
|  | 4.4. Is the risk of nonresponse bias low? |
|  | 4.5. Is the statistical analysis appropriate to answer the research question? |
| 5.Mixed methods | 5.1. Is there an adequate rationale for using a mixed methods design to address the research question? |
|  | 5.2. Are the different components of the study effectively integrated to answer the research question? |
|  | 5.3. Are the outputs of the integration of qualitative and quantitative components adequately interpreted? |
|  | 5.4. Are divergences and inconsistencies between quantitative and qualitative results adequately addressed? |
|  | 5.5. Do the different components of the study adhere to the quality criteria of each tradition of the methods involved? |

**Supplementary material 5 Quality assessment of the included studies**

| Author and year | Screening | | | Qualitative studies quality criteria | | | | | Randomized controlled trials quality criteria | | | | | Non-randomized studies quality criteria | | | | | Quantitative descriptive studies quality criteria | | | | | Mixed methods studies quality criteria | | | | |
| --- | --- | --- | --- | --- | --- | --- | --- | --- | --- | --- | --- | --- | --- | --- | --- | --- | --- | --- | --- | --- | --- | --- | --- | --- | --- | --- | --- | --- |
|  | S1 | S2 | 1.1 | | 1.2 | 1.3 | 1.4 | 1.5 | 2.1 | 2.2 | 2.3 | 2.4 | 2.5 | 3.1 | 3.2 | 3.3 | 3.4 | 3.5 | 4.1 | 4.2 | 4.3 | 4.4 | 4.5 | 5.1 | 5.2 | 5.3 | 5.4 | 5.5 |
| Liu and Wang (2025) | √ | √ | √ | | √ | × | √ | √ |  |  |  |  |  |  |  |  |  |  |  |  |  |  |  |  |  |  |  |  |
| Lee and Lee (2021) | √ | √ | √ | | √ | × | √ | × |  |  |  |  |  |  |  |  |  |  |  |  |  |  |  |  |  |  |  |  |
| Chen and Ping (2025) | √ | √ |  | |  |  |  |  | √ | √ | × | × | × |  |  |  |  |  |  |  |  |  |  |  |  |  |  |  |
| Guo and Xiong (2025) | √ | √ | √ | | √ | × | × | √ |  |  |  |  |  |  |  |  |  |  |  |  |  |  |  |  |  |  |  |  |
| Jia et al. (2026) | √ | √ | √ | | × | √ | √ | √ |  |  |  |  |  | √ | × | √ | √ | √ |  |  |  |  |  | √ | √ | √ | √ | × |
| Ye and Duan (2024) | √ | √ | √ | | √ | × | × | √ |  |  |  |  |  |  |  |  |  |  |  |  |  |  |  |  |  |  |  |  |
| Bofill-Herrero et al. (2025) | √ | √ | √ | | √ | √ | √ | √ |  |  |  |  |  |  |  |  |  |  |  |  |  |  |  |  |  |  |  |  |
| Hu and Min (2025) | √ | √ | √ | | √ | × | × | √ |  |  |  |  |  |  |  |  |  |  |  |  |  |  |  |  |  |  |  |  |
| Yin and Tang (2025) | √ | √ | √ | | √ | √ | √ | √ |  |  |  |  |  |  |  |  |  |  |  |  |  |  |  |  |  |  |  |  |
| Zhang et al. (2025) | √ | √ |  | |  |  |  |  |  |  |  |  |  |  |  |  |  |  | √ | √ | √ | × | √ |  |  |  |  |  |
| Wang et al. (2024) | √ | √ | √ | | √ | × | √ | × |  |  |  |  |  |  |  |  |  |  |  |  |  |  |  |  |  |  |  |  |
| Guo (2024) | √ | √ | √ | | √ | × | × | √ |  |  |  |  |  |  |  |  |  |  |  |  |  |  |  |  |  |  |  |  |
| Cao et al. (2025) | √ | √ | √ | | √ | × | √ | × |  |  |  |  |  |  |  |  |  |  |  |  |  |  |  |  |  |  |  |  |
| Duan (2025) | √ | √ | √ | | √ | √ | √ | × |  |  |  |  |  |  |  |  |  |  |  |  |  |  |  |  |  |  |  |  |
| Zhou et al. (2025) | √ | √ | √ | | √ | × | √ | × |  |  |  |  |  |  |  |  |  |  |  |  |  |  |  |  |  |  |  |  |
| Wang and Zhao (2025) | √ | √ | √ | | √ | √ | √ | √ |  |  |  |  |  |  |  |  |  |  |  |  |  |  |  |  |  |  |  |  |
| Li et al. (2024) | √ | √ |  | |  |  |  |  |  |  |  |  |  |  |  |  |  |  | √ | √ | √ | √ | √ |  |  |  |  |  |
| Dong and Liu (2024) | √ | √ | √ | | √ | √ | × | × |  |  |  |  |  |  |  |  |  |  |  |  |  |  |  |  |  |  |  |  |
| Martín-Rodríguez and Madrigal-Cerezo (2025) | √ | √ | √ | | × | × | √ | × |  |  |  |  |  |  |  |  |  |  |  |  |  |  |  |  |  |  |  |  |
| Chen et al. (2026) | √ | √ | √ | | √ | √ | √ | √ |  |  |  |  |  | × | √ | √ | × | √ |  |  |  |  |  | √ | √ | √ | √ | × |
| Zhang et al. (2024) | √ | √ | √ | | √ | × | × | √ |  |  |  |  |  |  |  |  |  |  |  |  |  |  |  |  |  |  |  |  |
| Yuan et al. (2024) | √ | √ | √ | | × | × | √ | × |  |  |  |  |  |  |  |  |  |  |  |  |  |  |  |  |  |  |  |  |
| Cui et al. (2026) | √ | √ | √ | | √ | √ | √ | √ |  |  |  |  |  |  |  |  |  |  |  |  |  |  |  |  |  |  |  |  |
| Chang et al. (2025) | √ | √ |  | |  |  |  |  |  |  |  |  |  | × | √ | × | × | √ |  |  |  |  |  |  |  |  |  |  |
| Konukman et al. (2025) | √ | √ | √ | | √ | × | × | × |  |  |  |  |  |  |  |  |  |  |  |  |  |  |  |  |  |  |  |  |
| Genç (2023) | √ | √ | √ | | × | √ | √ | × |  |  |  |  |  |  |  |  |  |  |  |  |  |  |  |  |  |  |  |  |
| Albaloul et al. (2024) | √ | √ | √ | | √ | × | √ | × |  |  |  |  |  |  |  |  |  |  |  |  |  |  |  |  |  |  |  |  |
| Guo and Xiong (2025) | √ | √ | √ | | √ | √ | √ | × |  |  |  |  |  |  |  |  |  |  |  |  |  |  |  |  |  |  |  |  |
| Wu and Jin (2024) | √ | √ | √ | | √ | × | √ | × |  |  |  |  |  |  |  |  |  |  |  |  |  |  |  |  |  |  |  |  |
| Dergaa et al. (2023) | √ | √ | √ | | × | × | √ | × |  |  |  |  |  |  |  |  |  |  |  |  |  |  |  |  |  |  |  |  |
| Killian et al. (2023) | √ | √ | √ | | √ | √ | × | × |  |  |  |  |  |  |  |  |  |  |  |  |  |  |  |  |  |  |  |  |
| Jiang et al. (2026) | √ | √ | √ | | √ | × | × | √ |  |  |  |  |  |  |  |  |  |  |  |  |  |  |  |  |  |  |  |  |
| Li et al. (2023) | √ | √ | √ | | √ | × | × | √ |  |  |  |  |  |  |  |  |  |  |  |  |  |  |  |  |  |  |  |  |
| Cheng and Wang (2025) | √ | √ | √ | | √ | × | √ | × |  |  |  |  |  |  |  |  |  |  |  |  |  |  |  |  |  |  |  |  |
| Zhou and Cheng (2021) | √ | √ | √ | | √ | × | × | × |  |  |  |  |  |  |  |  |  |  |  |  |  |  |  |  |  |  |  |  |
| Du and Qin (2025) | √ | √ | √ | | √ | × | √ | × |  |  |  |  |  |  |  |  |  |  |  |  |  |  |  |  |  |  |  |  |
| Baena-Morales et al. (2025) | √ | √ |  | |  |  |  |  |  |  |  |  |  | × | √ | × | × | √ |  |  |  |  |  |  |  |  |  |  |
| Wang (2023) | √ | √ | √ | | √ | × | × | × |  |  |  |  |  |  |  |  |  |  |  |  |  |  |  |  |  |  |  |  |
| Yang and Wang (2023) | √ | √ | √ | | √ | × | √ | × |  |  |  |  |  |  |  |  |  |  |  |  |  |  |  |  |  |  |  |  |
| Yang and Ren (2020) | √ | √ | √ | | √ | √ | √ | × |  |  |  |  |  |  |  |  |  |  |  |  |  |  |  |  |  |  |  |  |
| Liao et al. (2025) | √ | √ | √ | | √ | × | × | × |  |  |  |  |  |  |  |  |  |  |  |  |  |  |  |  |  |  |  |  |
| Zhang et al. (2025) | √ | √ | √ | | √ | × | √ | √ |  |  |  |  |  |  |  |  |  |  |  |  |  |  |  |  |  |  |  |  |
| Liu et al. (2023) | √ | √ | √ | | √ | × | × | √ |  |  |  |  |  |  |  |  |  |  |  |  |  |  |  |  |  |  |  |  |
| Gamlem et al. (2026) | √ | √ | √ | | √ | √ | √ | √ |  |  |  |  |  |  |  |  |  |  | √ | × | √ | × | √ | √ | √ | √ | √ | × |
| Feng and Zhao (2025) | √ | √ | √ | | √ | × | √ | √ |  |  |  |  |  |  |  |  |  |  |  |  |  |  |  |  |  |  |  |  |
| Wang (2025) | √ | √ | √ | | √ | × | × | √ |  |  |  |  |  |  |  |  |  |  |  |  |  |  |  |  |  |  |  |  |
| Wang and Dong (2024) | √ | √ | √ | | √ | × | √ | √ |  |  |  |  |  |  |  |  |  |  |  |  |  |  |  |  |  |  |  |  |
| Guo et al. (2025) | √ | √ | √ | | √ | × | √ | × |  |  |  |  |  |  |  |  |  |  |  |  |  |  |  |  |  |  |  |  |
| Lü and Tang (2024) | √ | √ | √ | | √ | × | √ | √ |  |  |  |  |  |  |  |  |  |  |  |  |  |  |  |  |  |  |  |  |
| Qiu and Zhang (2026) | √ | √ | √ | | √ | × | × | × |  |  |  |  |  |  |  |  |  |  |  |  |  |  |  |  |  |  |  |  |
| Ha et al. (2024) | √ | √ | √ | | √ | × | √ | √ |  |  |  |  |  |  |  |  |  |  |  |  |  |  |  |  |  |  |  |  |
| Yu and Wang (2025) | √ | √ | √ | | √ | √ | × | √ |  |  |  |  |  |  |  |  |  |  |  |  |  |  |  |  |  |  |  |  |
| Tannoubi et al. (2026) | √ | √ | √ | | √ | × | × | √ |  |  |  |  |  |  |  |  |  |  |  |  |  |  |  |  |  |  |  |  |
| Xu (2026) | √ | √ | √ | | √ | × | × | × |  |  |  |  |  |  |  |  |  |  |  |  |  |  |  |  |  |  |  |  |
| Wu et al. (2026) | √ | √ | √ | | √ | √ | √ | √ |  |  |  |  |  |  |  |  |  |  | √ | × | √ | × | √ | √ | √ | √ | √ | × |
| Guo et al. (2026) | √ | √ | √ | | √ | × | × | √ |  |  |  |  |  |  |  |  |  |  |  |  |  |  |  |  |  |  |  |  |
| Sun (2023) | √ | √ | √ | | √ | × | √ | × |  |  |  |  |  |  |  |  |  |  |  |  |  |  |  |  |  |  |  |  |
| Tao and Wang (2025) | √ | √ | √ | | √ | × | √ | √ |  |  |  |  |  |  |  |  |  |  |  |  |  |  |  |  |  |  |  |  |
| Xiao and Guo (2026) | √ | √ | √ | | √ | √ | √ | √ | √ | √ | √ | √ | √ |  |  |  |  |  |  |  |  |  |  | √ | √ | √ | √ | √ |
| Yang et al. (2025) | √ | √ |  | |  |  |  |  |  |  |  |  |  | × | √ | × | × | √ |  |  |  |  |  |  |  |  |  |  |
| Huang and Wang (2024) | √ | √ | √ | | √ | × | √ | × |  |  |  |  |  |  |  |  |  |  |  |  |  |  |  |  |  |  |  |  |
| Li et al. (2025) | √ | √ | √ | | √ | × | × | × |  |  |  |  |  |  |  |  |  |  |  |  |  |  |  |  |  |  |  |  |
| Yan (2025) | √ | √ | √ | | √ | × | × | √ |  |  |  |  |  |  |  |  |  |  |  |  |  |  |  |  |  |  |  |  |
| Østerlie and Killian (2026) | √ | √ | √ | | √ | × | √ | √ |  |  |  |  |  |  |  |  |  |  |  |  |  |  |  |  |  |  |  |  |
| Wang and Wang (2024) | √ | √ | √ | | × | × | √ | × |  |  |  |  |  |  |  |  |  |  |  |  |  |  |  |  |  |  |  |  |
| Can and Alas (2025) | √ | √ |  | |  |  |  |  |  |  |  |  |  | × | √ | × | × | √ |  |  |  |  |  |  |  |  |  |  |
| Yang (2022) | √ | √ | √ | | √ | × | √ | √ |  |  |  |  |  |  |  |  |  |  |  |  |  |  |  |  |  |  |  |  |
| Jiang et al. (2024) | √ | √ | √ | | √ | × | √ | × |  |  |  |  |  |  |  |  |  |  |  |  |  |  |  |  |  |  |  |  |
| Zhao and Xi (2023) | √ | √ | √ | | √ | × | √ | √ |  |  |  |  |  |  |  |  |  |  |  |  |  |  |  |  |  |  |  |  |
| Vahdani et al. (2026) | √ | √ | √ | | √ | × | × | √ |  |  |  |  |  |  |  |  |  |  |  |  |  |  |  |  |  |  |  |  |
| Cheng and Wang (2020) | √ | √ | √ | | √ | × | √ | × |  |  |  |  |  |  |  |  |  |  |  |  |  |  |  |  |  |  |  |  |
| Bergdahl and Sjöberg (2025) | √ | √ |  | |  |  |  |  |  |  |  |  |  |  |  |  |  |  | √ | × | √ | × | √ |  |  |  |  |  |
| Yu and Wang (2024) | √ | √ | √ | | √ | × | × | √ |  |  |  |  |  |  |  |  |  |  |  |  |  |  |  |  |  |  |  |  |
| Li et al. (2025) | √ | √ | √ | | √ | × | × | √ |  |  |  |  |  |  |  |  |  |  |  |  |  |  |  |  |  |  |  |  |
| Zamani and Pourranjbar (2026) | √ | √ | √ | | √ | √ | √ | √ |  |  |  |  |  |  |  |  |  |  |  |  |  |  |  |  |  |  |  |  |
| Wang and Cheng (2025) | √ | √ | √ | | √ | × | √ | √ |  |  |  |  |  |  |  |  |  |  |  |  |  |  |  |  |  |  |  |  |
| Xu et al. (2024) | √ | √ | √ | | √ | × | × | √ |  |  |  |  |  |  |  |  |  |  |  |  |  |  |  |  |  |  |  |  |
| Jiang et al. (2023) | √ | √ | √ | | √ | × | √ | × |  |  |  |  |  |  |  |  |  |  |  |  |  |  |  |  |  |  |  |  |
| Jiang et al. (2025) | √ | √ | √ | | √ | × | × | √ |  |  |  |  |  |  |  |  |  |  |  |  |  |  |  |  |  |  |  |  |
| Wang et al. (2024) | √ | √ |  | |  |  |  |  |  |  |  |  |  | √ | √ | √ | × | × |  |  |  |  |  |  |  |  |  |  |
| Wang (2024) | √ | √ | √ | | √ | × | √ | × |  |  |  |  |  |  |  |  |  |  |  |  |  |  |  |  |  |  |  |  |
| Li and Huang (2025) | √ | √ | √ | | √ | × | √ | × |  |  |  |  |  |  |  |  |  |  |  |  |  |  |  |  |  |  |  |  |
| Jiang et al. (2025) | √ | √ | √ | | √ | √ | √ | × |  |  |  |  |  |  |  |  |  |  |  |  |  |  |  |  |  |  |  |  |
| Ma and Mu (2025) | √ | √ | √ | | √ | × | √ | √ |  |  |  |  |  |  |  |  |  |  |  |  |  |  |  |  |  |  |  |  |
| Li et al. (2025) | √ | √ | √ | | √ | × | √ | × |  |  |  |  |  |  |  |  |  |  |  |  |  |  |  |  |  |  |  |  |
| Li et al. (2026) | √ | √ |  | |  |  |  |  |  |  |  |  |  | × | √ | √ | × | √ |  |  |  |  |  |  |  |  |  |  |
| Qin et al. (2025) | √ | √ | √ | | √ | √ | √ | × |  |  |  |  |  |  |  |  |  |  |  |  |  |  |  |  |  |  |  |  |
| Liu et al. (2024) | √ | √ | √ | | √ | × | × | √ |  |  |  |  |  |  |  |  |  |  |  |  |  |  |  |  |  |  |  |  |
| Zhang (2021) | √ | √ |  | |  |  |  |  |  |  |  |  |  | × | √ | √ | × | √ |  |  |  |  |  |  |  |  |  |  |
| Liao (2025) | √ | √ | √ | | √ | × | √ | × |  |  |  |  |  |  |  |  |  |  |  |  |  |  |  |  |  |  |  |  |
| Ouyang and Du (2023) | √ | √ | √ | | × | × | √ | × | × | × | × | × | √ |  |  |  |  |  |  |  |  |  |  | √ | √ | √ | √ | × |
| Ompoc and Aguinaldo (2025) | √ | √ |  | |  |  |  |  |  |  |  |  |  | × | √ | √ | √ | × |  |  |  |  |  |  |  |  |  |  |

Note: “√” stands for “Yes”, “×” stands for “No”, and “?” stands for “Can’t tell”.

# Supplementary material 6 Basic information on coding system (included the technology, physical education, and body dimension)

| Main categories | Preliminary categories | Concepts | Original statements |
| --- | --- | --- | --- |
| Technology | Data | Data leakage | The absence of robust data encryption and access control mechanisms increases the risk of student sports data leakage (Duan, 2025). The subcategory ‘Privacy and Security Concerns’ received 50 mentions (100%), reflecting students’ significant worries about data handling in AI-integrated PE (Bofill-Herrero et al., 2025). |
|  |  | Privacy infringement | Scientific digital assessment in physical education requires the collection of multimodal data (e.g., text, audio, images, and videos), but the widespread deployment of surveillance and audio-recording devices may infringe upon the privacy of teachers and students (Yuan et al., 2024). |
|  | Algorithm | Algorithmic bias | Intelligent physical education may give rise to technical and ethical risks, including data privacy invasion, data inaccuracies, and algorithmic bias (Wang et al., 2024). Algorithmic bias that may disadvantage certain groups of learners (Vahdani et al., 2026). |
|  |  | Algorithmic limitations | AI technology is diﬃcult to capture students’ emotional ﬂuctuations and cannot empathize with students, while emotional motivation plays an important role in PE (Wang and Wang, 2024). It is noted that the teaching of AI lacks cultural values, historical and social norms, and is deprived of abilities such as gesture, facial expression, and tone of voice (Can and Alas, 2025). |
| Physical education | Physical education teaching | Homogeneous physical education teaching | Although AI-driven personalized instruction aims to accommodate individual differences, it may evolve into a constrained form of standardization driven by technological logic, thereby limiting its flexibility in addressing students’ diverse needs (Liu and Wang, 2025). |
|  |  | Threats to teachers’ professional roles and agency | Otherwise, the high ability level offered by an AI PE teacher may result in PE teaching as a human profession disappear (Lee and Lee, 2021). |
|  |  | Deviation from the goals of physical education | AI has an inherent tendency to reinforce the logic that “what is measurable is valuable,” thereby contributing to the alienation of educational goals and the displacement of the value-oriented functions of physical education to some extent (Liu and Wang, 2025). While the platform enhances efficiency and personalization, it risks reducing the social and emotional benefits of traditional PE, such as peer collaboration and instructor-student rapport (Chen and Ping, 2025). |
|  | Physical education learning | Homogenization of student development | Market-driven intelligent physical education programs tend toward standardized, replicable teaching templates. This homogenized temporal object erases the individual differences of students in physical education, and mistakenly casts students into the image of abstract, homogenized “consumers” (Guo and Xiong, 2025). |
|  | Teacher-student relationship | Alienation in teacher-student relationships | Most (77.78%) teachers believed that artificial intelligence would have a greater impact on the relationship between physical education teachers and students in the future (Jia et al., 2026). |
|  |  | Alienation in student-student relationships | AI may hinder authentic interactions among students and between students and real-world contexts (Ye and Duan 2024). Scepticism revolved around AI’s real-world value and fears that over-reliance on technology could undermine human interaction and skill development (Bofill-Herrero et al., 2025). |
|  | Humanistic value | Lack of humanistic care | The design of AI-enabled systems may prioritize the quantification of health, physical fitness, and motor skills while overlooking or diminishing the moral and humanistic dimensions of physical education (Guo and Xiong, 2025). |
|  |  | Value alienation | AI technologies rely heavily on programmed algorithms and training data, making their outputs susceptible to value-laden biases embedded in the data, such as those related to gender, culture, and race. These biases may, in turn, subtly shape the values of learners engaged in self-directed learning (Hu and Min, 2025). |
|  | Academic research | Academic misconduct | When students use DeepSeek to generate personalized course papers or assignments, the absence of effective content detection mechanisms may also give rise to concerns regarding academic integrity (Yin and Tang, 2025). |
| Body | Biological Body | Blurred body boundary | AI technologies may blur bodily boundaries (Huang and Wang 2024). Although applications such as wearable devices and brain–computer interfaces can improve the efficiency of physical health assessment, they may also weaken students’ sense of bodily identity, bodily awareness, and capacity for self-reflection, potentially contributing to the objectification and instrumentalization of the body among adolescents (Cui et al. 2026). |
|  | Spiritual Body | Body meaning deconstruction | Technologies may deepen bodily awareness, but they may also mask or distort it by privileging data over sensation or norm-driven models over personal meaning (Østerlie & Killian, 2026). Excessive dependence on technology, it has been said, may lessen the need for physical exercise and engagement in physical education and sports, thereby harming the entire experience (Genç, 2023). |
|  |  | Body value alienation | If the use of technology evolves into misuse or excessive dependence, it may contribute to bodily deterioration and diminished physical capacity (Huang and Wang 2024). If ChatGPT generates inaccurate or misleading information without access to a user’s medical history, physical limitations, and other individualized factors, ChatGPT could negatively impact their health conditions (Ha et al., 2024). |

**Supplementary material 7 Number of included studies (n = 92) by journal**

| Rank | Journal | n |
| --- | --- | --- |
| 1 | Journal of Shenyang Sport University | 13 |
| 2 | Journal of Sports Research | 6 |
| 3 | Sports Culture Guide | 6 |
| 4 | China Sport Science | 5 |
| 5 | Journal of Physical Education | 4 |
| 6 | Journal of Shanghai University of Sport | 4 |
| 7 | The Journal of Physical Education, Recreation & Dance | 3 |
| 8 | Journal of Xi’an Physical Education University | 3 |
| 9 | Journal of Beijing Sport University | 3 |
| 10 | Journal of Chengdu Sport University | 3 |
| 11 | Sports & Science | 3 |
| 12 | Journal of Guangzhou Sport University | 2 |
| 13 | Frontiers in Public Health | 2 |
| 14 | Scientific Reports | 2 |
| 15 | Journal of Hunan University of Science and Technology (Social Science Edition) | 1 |
| 16 | Journal of Tianjin University of Sport | 1 |
| 17 | Journal of Teaching in Physical Education | 1 |
| 18 | Mediterranean Journal of Sport Science | 1 |
| 19 | Sustainability | 1 |
| 20 | Journal of Intelligent & Fuzzy Systems | 1 |
| 21 | Shanghai Research on Education | 1 |
| 22 | Teaching and Management | 1 |
| 23 | Education Academic Monthly | 1 |
| 24 | Journal of Wuhan Sports University | 1 |
| 25 | Theory and Practice of Education | 1 |
| 26 | Higher Education Exploration | 1 |
| 27 | Journal of Physical Education & Sport | 1 |
| 28 | Biology of Sport | 1 |
| 29 | International Journal on Semantic Web and Information Systems | 1 |
| 30 | Journal of Capital University of Physical Education and Sports | 1 |
| 31 | Journal of Shandong Sport University | 1 |
| 32 | Teaching & Administration | 1 |
| 33 | Physical Education and Sport Pedagogy | 1 |
| 34 | Computers and Education: Artificial Intelligence | 1 |
| 35 | Sport, Education and Society | 1 |
| 36 | Acta Psychologica | 1 |
| 37 | Journal of Hospitality, Leisure, Sport & Tourism Education | 1 |
| 38 | International Journal of Healthcare Information Systems and Informatics | 1 |
| 39 | Education Sciences | 1 |
| 40 | International Journal of Human Movement and Sports Sciences | 1 |
| 41 | Teaching and Teacher Education | 1 |
| 42 | Journal of Visualized Experiments | 1 |
| 43 | Journal of Cloud Computing | 1 |
| 44 | Learning, Media and Technology | 1 |
| 45 | Journal of English for Academic Purposes | 1 |
| 46 | International Journal of Information and Education Technology | 1 |
| 47 | Research in Sport Management and Marketing | 1 |
